# Understanding Viscoelasticity of an Entangled Silicone Copolymer via Coarse-Grained Molecular Dynamics Simulations

**DOI:** 10.1021/acs.macromol.5c01192

**Published:** 2025-08-11

**Authors:** Weikang Xian, Amitesh Maiti, Andrew P. Saab, Ying Li

**Affiliations:** † Department of Mechanical Engineering, 5228 University of Wisconsin-Madison, Madison, Wisconsin 53706-1572, United States; ‡ 4578 Lawrence Livermore National Laboratory, Livermore, California 94550, United States

## Abstract

Entangled dynamics is important for understanding rheological properties of long-chain polymers. For entangled homopolymers, the classic tube-reptation model and its refinements have been successfully applied to quantify properties like diffusion coefficient and zero-rate viscosity. However, the application of such models to copolymers has been limited despite scientific and industrial importance. Here, we study the entangled melt dynamics of poly­(dimethyl-*co*-diphenyl)­siloxane random copolymer for a range of mean-composition-ratio ϕ of the diphenyl component via long-term molecular dynamics simulation with a recently developed coarse-grained model. We found that the segmental relaxation is heterogeneous at the monomeric level because of compositional fluctuations. However, at the chain-entanglement level and higher length scales, the viscoelastic response is homogeneous with compositional dependence only through the overall diphenyl fraction ϕ. The relaxation modulus of the entangled copolymer melt conforms to the Likhtman–McLeish model, and the viscosity predicted using our current coarse-grained parameters is in good quantitative agreement with experimental data.

## Graphical abstract

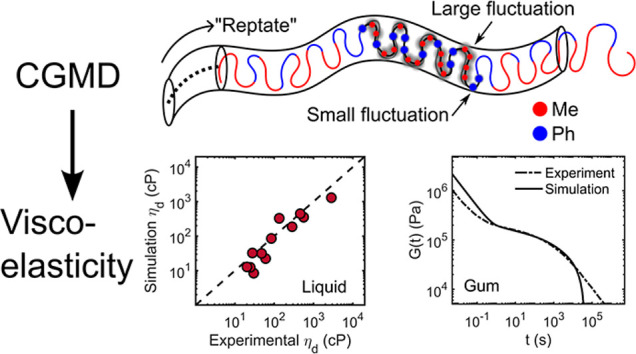

## 1 Introduction

Viscoelastic behaviors of polymer melts are governed by molecular relaxation of the constituent polymer chains that can be described as Gaussian random walk. The Rouse model quantifies the relaxation of unrestricted Brownian motion of short-chain molecules.^1^ However, for a melt of longer chains, topological noncrossability constraints, i.e., entanglements, lead to dynamics that is significantly restricted and thus accompanied by an increase in characteristic relaxation times. Proposed by de Gennes, Edwards, and Doi, such constraints effectively act as tube-like regions within which the chains “reptate” to relax.^2,3^ The tube-reptation model, together with later refinements including chain length fluctuation (CLF) and constraint release (CR) effects have been successfully used to quantify the dynamics of entangled polymer melt, such as the chain-length dependent diffusion coefficient and zero-rate viscosity that are important in scientific and industrial applications.^4^

Molecular dynamics (MD) has been widely recognized as an important tool for exploring the microscopic characteristics of polymeric materials.^5,6^ In particular, MD simulation allows one to investigate the structural and dynamic properties at length and time scales not easily accessible by experiments alone.^7^ While all-atomistic molecular dynamics (AAMD) is powerful in studying structural and dynamic properties of polymer materials at the monomeric and short-chain scales, coarse-grained molecular dynamics (CGMD) is more popular in the investigation of entangled dynamics because it overcomes the limitation of time and length scales of AAMD.^8^ For example, the seminal work by Kremer and Grest used the generic CGMD model to clarify the reptation-like molecular relaxation of the entangled system.^9^ Chemistry-specific CGMD models have also been developed to study the entangled dynamics of commodity homopolymers like polyethylene, polyisoprene, and polybutadiene.^10–13^ In fact, efforts based on these generic and chemistry-specific CGMD models greatly contribute to the current understanding of the effects of CLF and CR on the entangled dynamics. More recently, the CGMD models have also been used to quantify other effects like polydispersity and microscopic constraint.^14–16^ Additionally, mesoscale simulations are invaluable as they bridge the microscopic structure and dynamic properties of polymer materials to the macroscopic viscoelasticity. The Likhtman–McLeish (LM) model is one of the successful refinements of the tube-reptation model.^17^ It simplifies the molecular details of an entangled chain into fewer coarse-grained “links”. The effect of entanglement is modeled by virtual “springs” that are embedded in the thermodynamic background and evolved in time by “slipping” movement of the links. Other models have also been conceived based on the slip-link concept.^11,18–22^ It has been shown that these mesoscale scale models are powerful in predicting macroscopic viscoelasticity of polymer materials, such as nonlinear rate-dependent viscosity and end-retraction.^18,23^

However, these models usually assume that the chains are homopolymers with no variation in composition along the chain backbone. Thus, a natural question to ask is how applicable these models are for chains with heterogeneous topology or chemical functionality, as in copolymers? The great advantage of copolymer lies in the fact that the incorporation of even small amounts of chemical heterogeneity opens a vast design space that could be extremely beneficial.^24–26^ While diblock and triblock copolymers can exhibit varied structural patterns at micellar length scales, statistically random copolymers are promising for many novel designs,^27^ especially assisted by the fast-developing artificial intelligence and machine learning technologies.^28–30^

Although extensive experimental and theoretical studies have demonstrated the connection between the architecture and phase/morphology behavior of block copolymer, investigation of random copolymer seems less prevalent, despite its importance in numerous applications, with much attention paid to understanding the transition between ordered and disordered state.^31–35^ In terms of the molecular origins of the heterogeneity in dynamics, Lodge and McLeish provided a concise theoretical description of the self-concentration effect, a widely recognized concept that is used to relate the segmental relaxation to local environment.^36^ A prescribed length scale is needed to apply the Lodge–McLeish model. For block copolymer with incompatible blocks, assigning threshold of the length scale is straightforward because the time and length scales of the different blocks are usually well-separated. On the other hand, such a structure-based length scale to quantify the self-concentration effect seems less clear for random copolymers, although it is important for a quantitative understanding of the connection between the local and the bulk dynamics.

Polydimethylsiloxane (PDMS) is one of the most used silicone materials because of excellent mechanical, thermal, electrical, and biocompatible properties.^37^ Copolymerization with other polymers is a widely used strategy to improve the properties of PDMS.^38^ For example, copolymerization with methylphenyl or diphenyl siloxane significantly improves the thermal properties of PDMS, particularly its resistance to low-temperature-induced crystallization.^39–41^ It is found that the incorporation of the diphenyl components significantly alters the molecular relaxation and bulk viscoelasticity of the silicone materials.^41^ Previous AAMD simulations by Zhu et al. and by our group provided quantitative insights into the diphenyl-dependent structural and dynamic changes of the copolymers.^42,43^ However, the scope of such investigations was restricted to the unentangled regime due to limitations of total simulation times (<1 μs) that AAMD can afford.

To overcome these limitations, we developed a coarse-grained molecular dynamics (CGMD) model, which effectively leads to ∼3 orders-of-magnitude gain in simulation time and enables investigation of unentangled and slightly entangled melts of such copolymer systems.^44^ It is shown that the molecular relaxation of the copolymer systems is dependent on the mean-composition-ratio (ϕ) of the diphenyl monomers. In this work, macroscopic viscoelasticity of the entangled poly­(dimethyl-*co*-diphenyl)­siloxane (PDMS-*co*-PDPS) random copolymers is systematically investigated by long-term CGMD simulation, made possible by GPU-accelerated MD package HOOMD-blue.^45,46^ It is shown that the properties of the entangled copolymer system can be effectively sampled by the CGMD simulation, which can then be used to quantitatively predict the macroscopic viscoelastic properties that are consistent with experimental characterization. It is found that the molecular relaxation is homogeneous above the entanglement length scale. To that, we describe the computational and experimental methods in Section 2, while Section 3 details the results with a discussion. We summarize the important findings in Section 4.

## 2 Methods

### 2.1 Coarse-Grained Model

The structure and dynamics of the random copolymer systems significantly change with ϕ. For a particular system, ϕ is defined as the bulk ratio *N*_ph_/*N*, where *N* is the degree of polymerization of a linear molecule, and *N*_ph_ is the number of the diphenyl monomers of the same molecule. To study the ϕ-dependent properties of the PDMS-*co*-PDPS copolymers, we developed a chemistry-specific CGMD model, which effectively enables much longer time simulations than possible with the AAMD counterpart. We briefly review the CG model here, with more details available in our previous works.^44^ In this model, the dimethyl and diphenyl siloxane monomers of the linear chain are coarse-grained into CGB1 and CGB2 beads, respectively. The mapping scheme is shown in Figure 1a. Interactions of bond, angle, dihedral, and pair types are defined based on the topology of the copolymer. Potentials are optimized by the iterative Boltzmann inversion (IBI) method according to the structural distribution functions sampled from the ground-truth AAMD simulation.^43^ Polymer consistent force field (PCFF) is used for the AAMD simulation.^47^ Interestingly, we found that the nonbonded interactions are dependent on ϕ while the bonded interactions are independent of ϕ. As a result, the CG pair potential optimized based on a particular AA system is generally not applicable to other cases with different ϕ values. Therefore, a linear interpolation scheme is proposed to generate the pair potentials for the CG systems without AA reference. Detailed investigation shows that the interpolating rule can successfully preserve the radial distribution functions and other structural and dynamic properties reasonably well. Additionally, we developed an in-house protocol to generate initial CG configurations for the model systems. The protocol equilibrates a collection of random walks, with ϕ-dependent sequences of CGB1 and CGB2. In a soft-repulsion fashion,^48^ the equilibration gradually turns on the strength of the nonbonded interactions via a linear coefficient while maintaining the full strength of the bonded interactions. The incremental equilibration procedure is continued until both the fluctuations of the bond length and system temperature are within 5% of the target values. An additional relaxation MD run in isothermal–isobaric ensemble is conducted to ensure equilibrium of the model system. In the current study, the degrees of polymerization of the linear molecule, *N*, are 250, 400, 600, and 800, covering the range from slightly entangled to well-entangled states. The numbers of chains *n* are 250, 250, 220, and 200 for the respective *N* systems. The values of molar ratio ϕ are 0.0, 0.05, 0.15, 0.10, 0.20, and 0.40. Hereinafter, each system is labeled as CL­(*N*)­R­(100ϕ). For example, CL800R40 refers to the system with *N* = 800 and ϕ = 0.40. Simulations are performed on 24 different systems in total. The dynamics of all systems are mapped back to the physically relevant scale with corresponding ϕ-dependent time rescaling factors *S*_τ_ to compensate for the mismatch in dynamics between the AA and CG models.^44^ It is worth noting that one CGB1 bead lumps ten atoms for a dimethylsiloxane monomer, while one CGB2 bead lumps 24 atoms for a diphenyl siloxane monomer. Therefore, the time rescaling factor *S*_τ_ monotonically increases with ϕ as the coarse-graining level increases with ϕ. It is defined as *S*_τ_ = *D*_CG_/*D*_AA_, where *D*_CG_ and *D*_AA_ are the diffusion coefficients from the coarse-grained and all-atomistic simulations, respectively.^44^ The results are shown in Table S1 in the Supporting Information.

**Figure 1:**
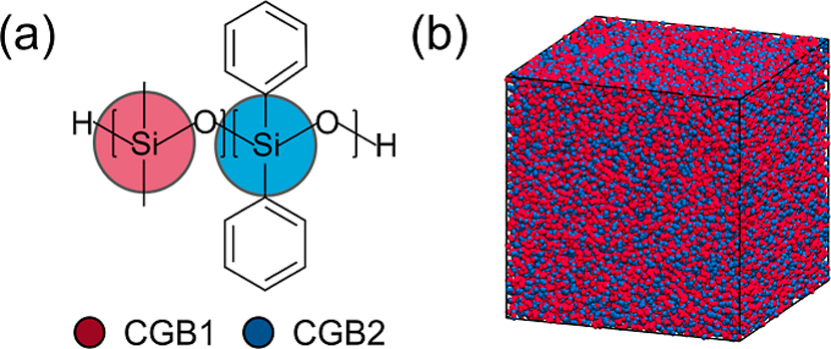
Coarse-grained model for PDMS-*co*-PDPS random copolymer. (a) The silicon atom of a siloxane monomer is chosen as the center of the CG bead. The derived CG beads are termed CGB1 and CGB2 for the dimethyl and the diphenyl monomers, respectively. (b) A snapshot of the CL800R40 system. Periodic boundary conditions are applied in all directions.

### 2.2 Molecular Dynamics Simulation

The CGMD simulations are conducted using the HOOMD-blue package that offers excellent GPU-acceleration.^45,46^ In these simulations, harmonic form is used for the covalently bonded interactions, while tabulated form is used for angle, dihedral, and pair interactions. All potential parameters are adapted from results of our previous work.^44^ The values of 0.3 and 2.5 nm are used for the neighbor list buffer and the cutoff, respectively. The production simulation is performed in the canonical ensemble. The Bussi–Donadio–Parrinello thermostat is used to regulate the temperature of the simulation system.^49^ A time step of 7.33 fs is used for the CL250 and CL400 systems, while 3.67 fs is used for the CL600 and CL800 systems to balance computational stability and efficiency. Choosing 3.67 or 7.33 fs for the time step does not affect the dynamic properties of the CL250 and CL400 systems, so the latter is used to reduce the computational cost. On the other hand, the smaller time step is used for the CL600 and CL800 systems to avoid numerical instability that is observed during the preproduction run, which may be related to an unknown GPU communication issue. Note that simulations for the CL250 and CL400 systems were performed using single-GPU jobs, whereas dual-GPU jobs were employed for the CL600 and CL800 simulations. The simulation temperature is set to 550 K, and the time constant for the thermostat is five times the assigned timesteps. It should be noted that pure polydiphenylsiloxane has a very high glass transition temperature *T*_g_ = 328.0 K and melting temperature *T*_m_ = 823.2 K.^50,51^ On the other hand, pure PDMS has values of *T*_g_ ∼ 150.0 K and *T*_m_ ∼ 210.0 K.^41^ The incorporation of the diphenyl composition prevents the copolymer from crystallizing. Additionally, the high simulation temperature ensures that the modeled systems are fully miscible. A snapshot of the CL800R40 system, with periodic boundary conditions applied in all directions, is shown in Figure 1b. The Visual Molecular Dynamics (VMD) package is used for visualization.^52^ An additional equilibration that lasts for 50 ns is applied to each system to avoid any possible negative effect caused by the format conversion. The mean-squared internal distance (MSID) curves are examined and the equilibrium for all systems is confirmed before the production run. See the Supporting Information for the definition of MSID. The MSID results are shown in Figure S1, where the plateau indicates that each system is sufficiently equilibrated. The longest production run lasts over 10 billion steps. The trajectory-dumping interval is 5000 and 10,000 steps for the time step choices of 7.33 and 3.67 fs, respectively. Shared hardware equipped with NVIDIA H100 GPU in the Krestrel cluster of National Renewable Energy Laboratory (NREL) and in-house hardware with the same specs in the Euler cluster of College of Engineering, University of Wisconsin–Madison were used for these production runs. Hardware equipped with NVIDIA V100 GPU in the Lassen cluster of Lawrence Livermore National Laboratory (LLNL) was used for auxiliary simulation. Version 4.8.2 of HOOMD-blue was used as the MD engine. Additionally, the multitau algorithm was implemented as a Custom Action in HOOMD-blue to calculate the relaxation modulus on-the-fly.^53^ Relaxations were tracked by the mean-squared displacement (MSD) to ensure that all model systems entered the diffusive regime.

### 2.3 Entanglement Analysis

Entanglement-related structural property is analyzed by the Z1 algorithm.^54,55^ The algorithm uses geometrical optimization to identify the kinks and minimum paths of the individual linear chains while enforcing the topological uncrossability of entangled chains. The number of kinks ⟨*Z*⟩, the primitive path length *L*_pp_, the classical entanglement length based on the kink number *N*_ck_ (classical kink definition), and the tube diameter *a*_pp_ are estimated.

### 2.4 Other Analyses

A key quantity to understand the dynamics of the entangled system is the surviving tube fraction μ­(*t*), i.e., the fraction of the primitive path that remains within the original tube (formed at *t* = 0) at time *t*.^2,56,57^ The tube-reptation model predicts, as in eq 1, that

1 $$\mu \left(t\right)=\frac{8}{\pi^{2}}\sum_{p,odd}^{\infty }\frac{1}{p^{2}}\exp\left(-\frac{p^{2}t}{\tau_{p=1}}\right)$$

where τ_*p*_ is the characteristic relaxation time. On the other hand, the prediction of the LM model is given in eq 2

2 $$\mu \left(t\right)=\int_{\varepsilon^{*}\left(Z,\tau_{e}\right)}^{\infty }\frac{0.306}{Z\tau_{e}^{1/4}\varepsilon^{4/5}}\exp\left(\varepsilon t\right)d\varepsilon +\frac{8{\mathrm{G̃}}_{f}\left(Z\right)}{\pi^{2}}\sum_{p,odd}^{p^{*}\left(Z\right)}\frac{1}{p^{2}}\exp\left(-\frac{p^{2}t}{\tau_{df}\left(Z,\tau_{e}\right)}\right)$$

To explicitly quantify such evolution in the MD simulation, a topological analysis based on the primitive path configurations obtained by the Z1 algorithm is performed to estimate the segment survival probability ψ­(*s*,*t*), i.e., the probability that the tube segment *s* remains in the original tube at *t*.^58^ A quantity closely related to μ­(*t*) is the tube survival probability Ψ­(*t*) as defined by eq 3

3 $$\Psi \left(t\right)=\frac{1}{L}\int_{0}^{L}\psi \left(s,t\right)ds$$

where *s* is the curvilinear coordinate. Additionally, it is proposed by the tube-reptation model that μ­(*t*) is equivalent to the normalized autocorrelation function as in eq 4

4 P(t)=⟨R(t)·R(0)⟩/⟨R(0)·R(0)⟩

where ***R*** is the end-to-end vector for a linear chain, although the equivalency does not hold if the CLF and CR effects are considered. Additional metrics are also used to track the dynamics of the copolymer system. The monomeric motion in space is quantified by the mean-squared displacement (MSD) as in eq 5

5 $$\begin{array}{l} g_{1}\left(t\right)=\left\langle {\left[r\left(t\right)-r\left(0\right)\right]}^{2}\right\rangle \\ g_{2}\left(t\right)=\left\langle {\left[r\left(t\right)-r_{CM}\left(t\right)-r\left(0\right)+r_{CM}\left(0\right)\right]}^{2}\right\rangle \\ g_{3}\left(t\right)=\left\langle {\left[r_{CM}\left(t\right)-r_{CM}\left(0\right)\right]}^{2}\right\rangle \end{array}$$

where ***r*** is the coordinate of a CG bead. *g*_3_(*t*) and *g*_2_(*t*) are defined for the center-of-mass (CM) motion and for the monomeric motion relative to the chain-CM, respectively. The scaling behaviors of the MSD are useful to distinguish different regimes during the relaxation processes. For example, the scaling of *g*_1_ ∼ *t* and *g*_3_ ∼ *t* indicate Fickian diffusion. The Fickian diffusion coefficient is calculated according to *D* = *g*_3_/6*t*. Moreover, the normal-mode analysis (NMA) is used to quantify the decoupled relaxation processes of the polymer chains. The NMA essentially transforms the Cartesian coordinate, ***r***, of a chain to the orthonormal counterpart, ***X***, as in eq 6

6 $$X_{p}=\frac{1}{N}\sum_{j=1}^{N}\left[\cos\frac{\pi p}{N}\left(j-\frac{1}{2}\right)\right]r_{j}$$

where *p* is the mode order. The autocorrelation function, *C*_*p*_(*t*) = ⟨ ***X***_*p*_(*t*)· ***X***_*p*_(0)⟩/⟨ ***X***_*p*_(0)· ***X***_*p*_(0)⟩, of the orthonormal coordinate is used to estimate the mode-dependent relaxation time, τ_*p*_, according to τ_*p*_ = τ_1_/*p* ^2^. The largest relaxation time, τ_1_, represents the time scale of full decorrelation of the conformation of a molecule. Another useful metric is the coherent dynamic structure factor (DSF), a single-chain quantity that is related to the observation of the neutron spin–echo spectroscopy experiment,^59–63^ which is calculated as in eq 7

7 $$S\left(q,t\right)=\frac{1}{N}\sum_{j,k}^{N}\left\langle \exp\left\{iq\cdot \left[r_{j}\left(t\right)-r_{k}\left(0\right)\right]\right\}\right\rangle$$

where ***q*** is the reciprocal wave vector. In isotropic cases, like all systems in this work, it is sufficient to quantify only the magnitude of ***S*** (***q***,*t*).

The relaxation modulus, *G*(*t*), is used to quantify the bulk viscoelastic characteristics. It is calculated by the Green–Kubo method,^64–66^ as in eq 8

8 $$G\left(t\right)=\frac{V}{k_{B}T}\left\langle \sigma_{ij}\left(t\right)\sigma_{ij}\left(0\right)\right\rangle ,\;i\neq j$$

where σ_*ij*_ is the off-diagonal element of the stress tensor σ. *k*_B_ *T* is the thermal energy, and *V* is the volume of the model system. The autocorrelation function within the angular bracket is calculated by the multitau algorithm^53^ and averaged over all possible pairs of *i* and *j*. During the CGMD simulation, stress data is recorded and fed into the multitau algorithm to maximize the usage of the stress data to facilitate calculating the long-time correlation.

Finally, the time-averaging trajectories are obtained based on the instantaneous trajectories from the CGMD simulation to analyze the local composition-dependent structural and dynamic properties. The MD trajectories within a time window, τ_ave_, are averaged according to eq 9

9 $$\mathrm{r̅}=\frac{1}{\tau_{ave}}\int_{-\tau_{ave}/2}^{\tau_{ave}/2}r\left(t\right)dt$$

A local Cartesian coordinate system is defined according to the average trajectory, within which the transverse and longitudinal displacements, *r*_⊥_ and *r*_∥_, respectively, are quantified by projecting the vector components of *r*–***r* ®** to the local coordinate system (see Section 3.7 for more details). The local composition, ϕ_loc_, is defined by the ratio of the number of CGB2 beads, i.e., diphenyl monomers, to the total number of beads in the neighbor that includes the adjacent 10 beads on both sides of the probed bead and the probed bead itself. The choice of 21 beads, over which ϕ_loc_ is averaged, is found to be appropriate to quantify the local heterogeneity. Such a choice ensures that the chosen segment has a size that is larger than a Kuhn segment but smaller than an entanglement segment so that the local composition is probed effectively. Note that a larger number of beads results in ϕ_loc_ not much different from ϕ, while a smaller number of beads leads to only a few discrete values of ϕ_loc_, which makes it statistically difficult to quantify the correlation between ϕ_loc_ and other properties. All these analyses were performed by in-house scripts.

### 2.5 Experimental Viscoelasticity

Liquid samples of the PDMS-*co*-PDMS linear random copolymers were purchased from Gelest. The materials include vinyl-terminated copolymers with different molecular weights and molar ratios ϕ. Trimethylsiloxy-terminated pure PDMS materials were also used. Table 1 lists the information on the materials, where η_k_ is the nominal kinematic viscosity at room temperature (278.0 K), and *M*_w_ is the weight-averaged molecular weight provided by the vendor. A Brookfield rotational viscometer was used to measure the steady-state dynamic viscosity η_d_ of the copolymer liquids. The materials were used as received. The degree-of-polymerization (*N*) values were converted according to ϕ and *M*_w_. The copolymer liquids were entangled. Depending on the material-specific viscosity, different rotors were paired with the cylindrical test-container, which can hold about 20.0 mL of liquid. The strain rates of measurement varied from 3.0 to 20.0 s^–1^, within the linear viscoelastic regime as they are much smaller than the inverse characteristic times of the materials (e.g., ≪1/τ_d_). The measurement temperature changed from 298.0 to 373.0 K. A 10 min equilibration was applied after each temperature change. At each temperature, viscosity results with at least five different strain rates were averaged. The viscometer was calibrated with viscosity standards from CANNON Instrument. An Arrhenius-like relation was used to extrapolate results of viscosity at 550.0 K since applying such high temperature could induce degradation of the copolymer. The original Arrhenius equation generally quantifies a reaction-rate *k*_c_ = *A* exp­(−*E*_a_/*k*_B_ *T*), where *E*_a_ is the activation energy barrier and *A* is a coefficient. It was assumed that ${\eta_{d}∼k}_{c}$ because of the facts that the viscoelastic behavior of the copolymer is governed by the microscopic friction and that the temperature-dependent viscosity is a macroscopic representation of the microscopic activation level. Therefore, the temperature-dependent results of η_d_ were fitted against log­(η_d_) = *aT* ^–1^ + *b* where *a* and *b* are fitting parameters. The fitting and extrapolation are shown in Figures S2–S5. The η_d_ results are summarized in Table 1. Additionally, the relaxation moduli of the two highly entangled copolymer gums were derived based on the rheological master curves from our previous study.^41^ The master curves were constructed according to the time–temperature superposition (TTS).^67^ Table 2 lists the material properties of the copolymer gums.

**Table 1:** Properties of Copolymer Liquids Purchased from Gelest^a^

	*M*_w_ (kg/mol)	mean-ratio ϕ	chain length *N*	η_k_ (cSt) 298 K	experiment η_d_ (cP) 550 K	prediction η_d_ (cP) 550 K
PDV0325	15.5	0.0325	198	500	24.4	12.4
PDV0331	27.0	0.0325	345	1000	47.5	31.4
PDV0346	78.0	0.0325	1010	60,000	2840.8	1290.4
PDV0525	14.0	0.05	174	500	20.2	12.7
PDV0535	47.5	0.05	591	5000	284.5	186.5
PDV0541	60.0	0.05	747	10,000	458.55	444.1
PDV1631	19.0	0.16	202	1000	27.7	32.3
PDV1635	35.3	0.16	375	5000	84.1	85.1
PDV1641	55.0	0.16	585	10,000	133.8	327.7
DMST25	17.3	0.0	233	500	29.9	8.4
DMST31	28.0	0.0	378	1000	60.2	22.2
DMST41	62.7	0.0	847	10,000	559.3	352.1

a There are 9 vinyl-terminated copolymers and 3 trimethylsiloxy-terminated pure PDMS homopolymers.

**Table 2:** Properties of Copolymer Gums Used for the Rheological Measurement^a^

	*M*_n_ (kg/mol)	*M*_w_ (kg/mol)	ϕ	*N*
LVM	320	680	0.035	4076
SE	450	880	0.053	5600

a There is less than 2% methylvinylsiloxane but not shown here.

For the experimental systems, simulation-based relaxation modulus curves were generated according to the LM model parametrized by the MD-based structural and dynamic properties. For the copolymer liquids, the relaxation modulus curves were used to derive the zero-rate viscosity according to η_d_ = ∫_0_^∞^ *G*(*t*)­d*t*. For the two copolymer gums, the generalized Maxwell model was used to fit the master curves (as shown in Figure S6), and the fitting results were used to calculate the relaxation modulus. The relaxation modulus results were converted to the reference temperature to compare with the results from the rheological measurements. Please refer to Supporting Information for more information.

## 3 Results

### 3.1 Mean-Squared Displacement

We use the *g*_1_ and *g*_3_ curves to confirm full relaxation of the simulation system. For example, the results for the CL250R0 and CL800R40 systems are shown in Figure 2a,b. Upon full relaxation, the *g*_1_ and *g*_3_ curves approach each other and increase linearly with *t*. The mean-squared end-to-end distance, ⟨*R*_ee_^2^⟩, is used to estimate the disentanglement time τ_d_ by equating *g*_3_(τ_d_) = ⟨*R*_ee_^2^⟩. The last points of the MSID curves, as shown in Figure S1, are chosen for the equilibrium values of ⟨*R*_ee_^2^⟩. The complete lists of MSD curves are shown in Figures S7–S10. As the relaxation enters into the diffusive regime, a plateau develops for the *g*_2_ curve, suggesting full relaxation. For some cases of the CL600 and CL800 systems, the *g*_3_ curves do not cross the respective ⟨*R*_ee_^2^⟩. Therefore, the τ_d_ of these cases are extrapolated by assuming *g*_3_∼*t*. The extrapolation sufficiently estimates the τ_d_ values as shown in the following sections. The rescaled *g*_1_/*t* ^1/2^ curves are used to estimate other characteristic times according to the scaling behavior of *g*_1_ curves, as shown in Figure 2c,d, where the CL250R0 and CL800R40 systems are used as examples. The MSD of the innermost 10% of the CG beads of the chains is used to calculate the rescaled *g*_1_ curves for better estimation of the tube-like behavior, which is a well-recognized practice.^58,68^ In Figure 2c,d, distinct behaviors of *g*_1_ in different regimes of relaxation are marked. The *g*_1_ curve shoots up at the beginning with a *g*_1_ ∼ *t* scaling (not labeled) and then is followed by a transition to *g*_1_ ∼ *t* ^1/2^ that is indicated by the first inflection zone, suggesting a Rouse-like behavior until the monomeric motion is hindered by the tube-like constraint. It is predicted that the tube-like constraint yields a *g*_1_ ∼ *t* ^1/4^ scaling, an asymptotic behavior that is not always observed.^9^ Therefore, fitting is performed to obtain the scaling index, and the values are shown in the figures. It is observed that systems with more entanglement (i.e., longer chain length) have scaling index values that are closer to the theoretical prediction of 1/4. By intersecting the two linear fittings for the Rouse and the tube-constraining regimes,^58^ the entanglement time, τ_e_, is identified, and the corresponding displacement is assigned as the tube radius. The values are used to calculate the segment survival probability ψ­(*s*,*t*). As the curvilinear diffusion proceeds, the transition from ∼*t* ^1/4^ to ∼*t* ^1/2^ behavior is expected as the tube primitive path itself is a random walk.^4^ The Rouse time, τ_R_, is identified by the intersecting points of the ∼*t* ^1/4^ to the second ∼*t* ^1/2^ linear fittings. In the last regime of relaxation, the *g*_1_ ∼ *t* behavior indicates Fickian diffusion. But the fitting result is shown only for visual purposes and not used for any analysis. In Figure 2e, the diffusion coefficient, *D*, is plotted as a function of the chain length *N*. The tube-reptation model predicts a *D* ∼ *N* ^2^ scaling behavior, and experimental observation follows an asymptotic *D* ∼ *N* ^2.4^ behavior for highly entangled systems with tens to hundreds of entanglements.^69^ However, the CGMD result shows intermediate behavior, which is attributed to the fact that the model systems cover the range from slightly entangled to well-entangled states but not to very highly entangled states. In Figure 2f, the diffusion coefficient is plotted with respect to the mean molar ratio ϕ, with a scaling of *D* ∼ 10^–2.7ϕ^, confirming that the CGMD simulation implemented in HOOMD is consistent with our previous works.^43,44^

**Figure 2:**
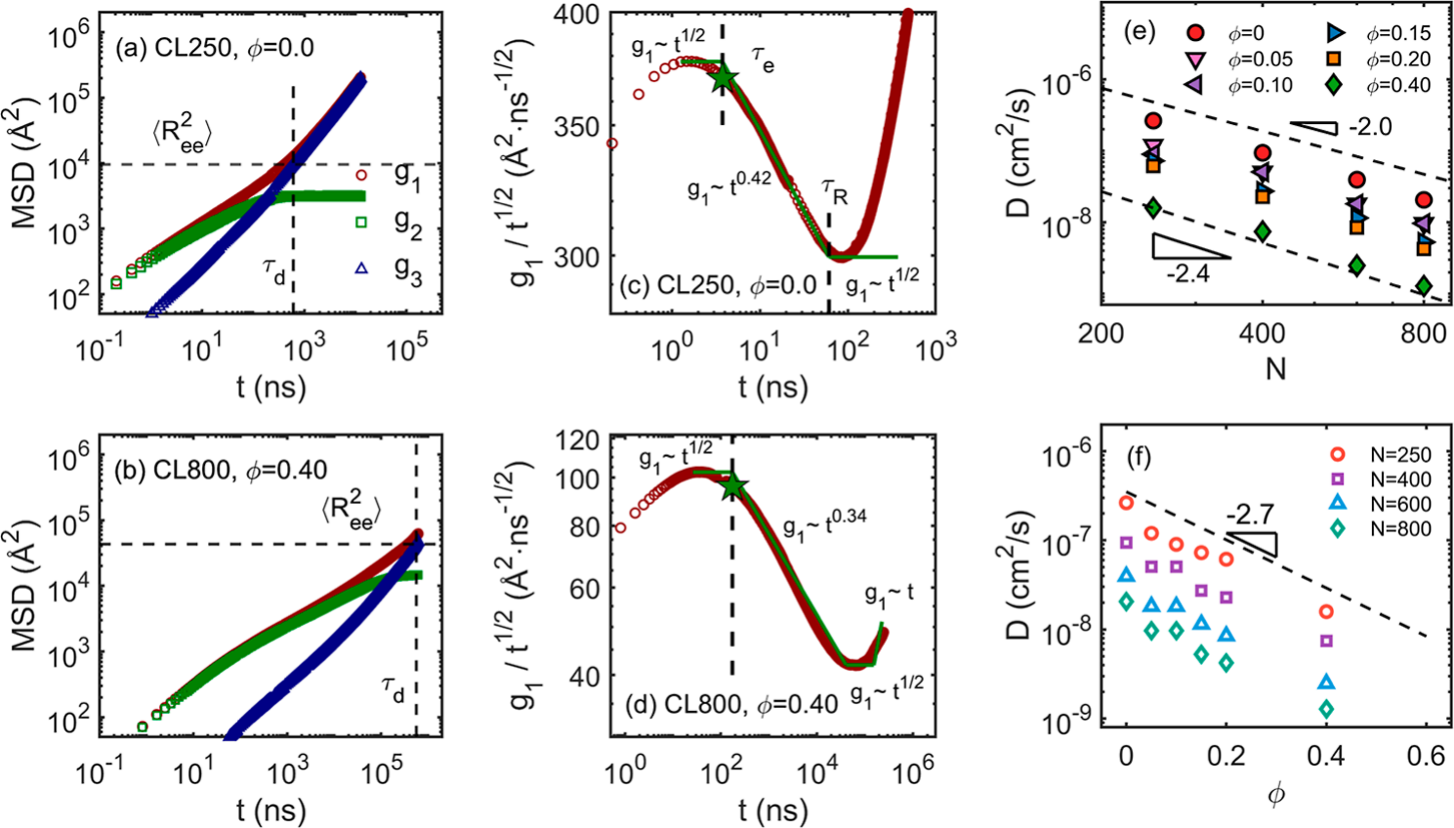
Mean-squared displacement. (a,b) are the MSD results for the CL250R0 and CL800R40 systems with the respective mean-squared end-to-end distance ⟨*R*_ee_^2^⟩ and disentanglement time τ_d_ marked. (c,d) are the rescaled *g*_1_ curves used to estimate the entanglement time τ_e_ and the Rouse time τ_R_, which are derived by intersecting the fittings for different regimes. The penta-star represents the tube radius *a*_pp_/2 estimated by the corresponding τ_e_. (e,f) are the diffusion coefficients *D* plotted as functions of the chain length *N* and the molar ratio ϕ, respectively.

### 3.2 Monomeric Relaxation Time

In the Rouse or the tube-reptation models, the smallest time scale is set by the monomeric relaxation time τ_0_, with three conceptually equivalent expressions: (1) τ_0_ = τ_e_ *N*_e_^–2^; (2) τ_0_ = τ_R_ *N* ^–2^; and (3) τ_0_ = τ_d_ *N*_e_ *N* ^–3^. The characteristic times τ_e_, τ_R_, and τ_d_ identified from the MSD results are shown in Figures S11 and used to calculate τ_0_. The τ_0_ values are plotted against ϕ in Figure 3. The AAMD values from our previous work are also included for a direct comparison.^43^ The CGMD result agrees quantitatively with the AAMD reference. In Figure 3a,b, the discrepancy is attributable to the fact that τ_e_ and τ_R_ are fitted with inevitable numeric uncertainty. Additionally, the estimation of the segmental dynamics may be inaccurate because the dynamics of the CGMD model is rescaled by comparing the diffusion coefficient of the CG 50-mers with their AA counterparts.^44^ The excellent agreement between the CGMD results and the AAMD reference in Figure 3c suggests that it is reasonable to estimate τ_d_ by the equality *g*_3_(τ_d_) = ⟨*R*_ee_^2^⟩ (or by extrapolation if needed) and to rescale the CGMD dynamics by comparing the diffusion coefficients.

**Figure 3:**
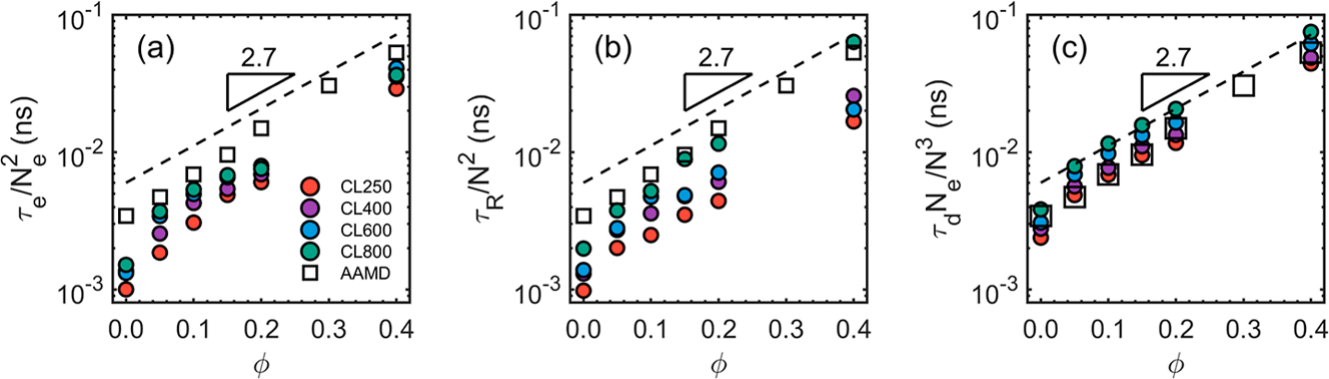
Estimation of the monomeric relaxation time τ_0_. The result of CL50 systems from our previous AAMD simulation work^43^ is included for a direct comparison. (a) τ_0_ is estimated by rescaling the entanglement time τ_e_. (b) τ_0_ by rescaling the Rouse time τ_R_. (c) τ_0_ by rescaling the disentanglement time τ_d_. In all cases, the scaling of τ_0_ ∼ 10^2.7ϕ^ is consistent with our previous works.^43,44^ The dashed line is guide-to-eye.

It is worth noting that the scaling behavior is not uniform across the entire range of ϕ. A close inspection suggests that the slope is larger than 2.7 when ϕ < 0.2. Similar deviation is also observed for the cases with ϕ < 0.2 in Figure 3. Such deviation highlights the importance of the current investigation as the CGMD model overcomes the limitation of time and length scales. However, extra effort is needed to quantify the deviation, and it is out of the scope of this work.

### 3.3 Entanglement Analysis

The structural characterization of entanglement is necessary for a more in-depth understanding of the relaxation dynamics. Figure 4a shows that the entanglement number ⟨*Z*⟩ increases nearly proportionally with the chain-length *N*, while the dependence on ϕ is weak, increasing slightly at ϕ = 0.40. In Figure 4b,c, the ϕ-dependence of the primitive path length *L*_pp_ and the entanglement length *N*_e_ = *N*_ck_ (classical kink definition) suggest that the chain stiffness and the level of entanglement increase with ϕ, which agrees well with the conclusion of our previous works.^44^ The decrease in *N*_ck_ at ϕ = 0.40 (Figure 4c) is a result of the corresponding increase in ⟨*Z*⟩ (Figure 4a). The entanglement molecular weights *M*_e_ based on *N*_ck_ are reported in Table 3. It is worth noting that our CGMD model predicts the entanglement structure well. The Z1 analysis of the CL800R0 system yields a tube diameter of *a*_pp_ = 7.5 nm and an entanglement length *N*_ck_ = 74.8, which agree well with experimental measurements of *a*_pp_ = 7.9 nm and *N*_rheo_ = 166.1, respectively.^70^ Note that *N*_rheo_ ≈ 2*N*_ck_ is expected.^71^

**Figure 4:**
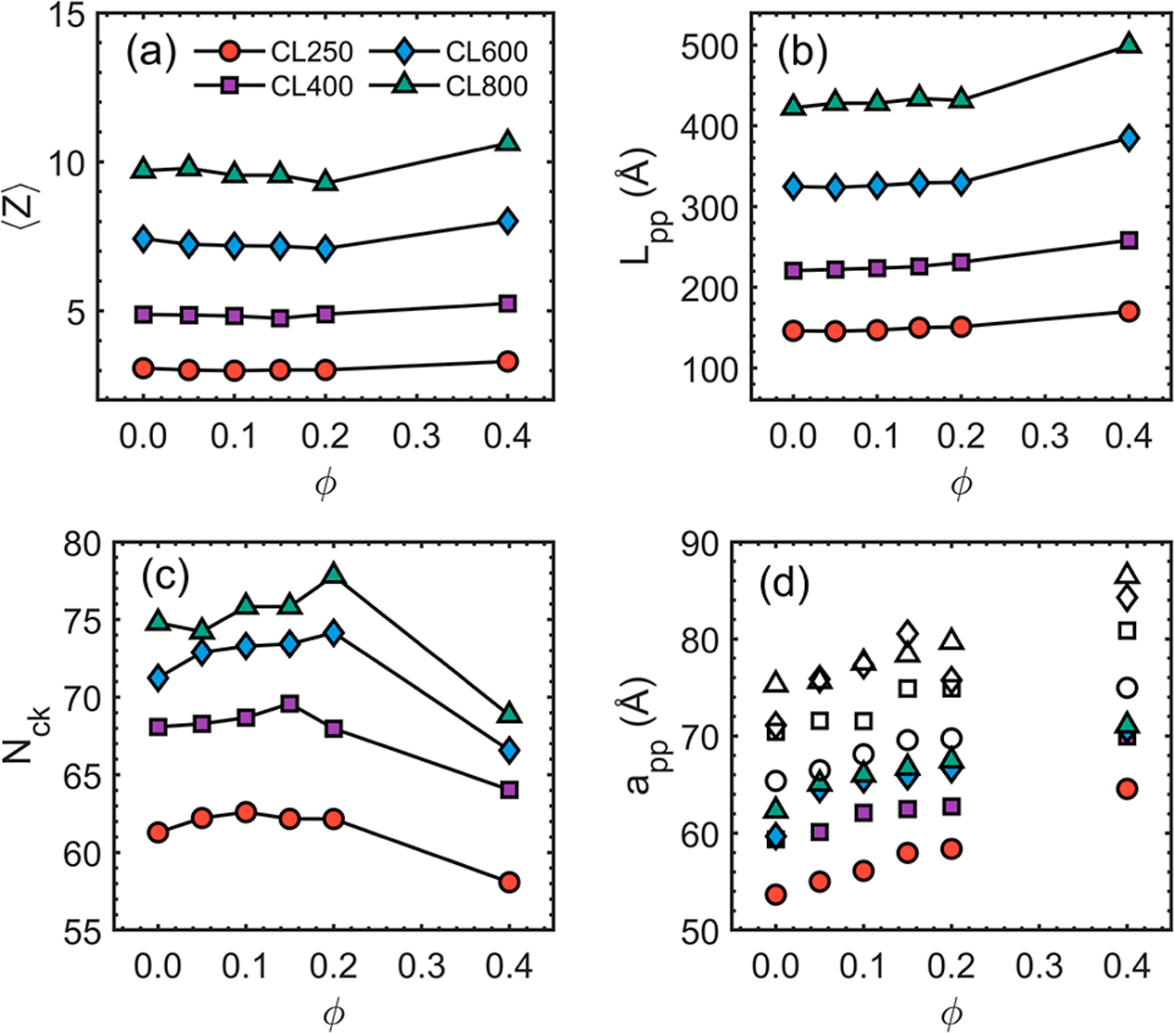
Entanglement analysis by Z1 algorithm. (a) Number of kinks (i.e., entanglement number) is plotted as functions of the molar ratio ϕ. (b) Primitive path length increases monotonically with the ϕ. (c) Entanglement length increases with the chain length *N*. But its dependence on ϕ is nonmonotonic. (d) Tube diameter values estimated by the MSD criteria and by the Z1 algorithm are in solid and hollow symbols, respectively.

**Table 3:** Estimation of Entanglement Molecular Weight (*M*_e_) for the Copolymer, Based on the Identified Entanglement Length *N*_ck_ from the Z1 Analysis

ϕ	0.0	0.05	0.1	0.15	0.2	0.4
*M*_e_ (kg/mol)	5.55	5.97	6.56	7.04	7.70	8.52

In Figure 4d, the tube diameters, *a*_pp_, estimated by the Z1 analysis, are shown with the values estimated by $2\sqrt{g_{1}\left(t_{e}\right)}$ (see Figure 1c,d). The discrepancy between the two definitions is attributed to the fact that *a*_pp_ is treated as the mean size of the entanglement segment in the Z1 analysis and in the classical tube theory. Such a length scale may not accurately reflect the constraint strength of the entanglement.^71–73^ On the other hand, the estimation by $2\sqrt{g_{1}\left(t_{e}\right)}$ probes the local monomeric motion subject to the entanglement constraint in a time-explicit fashion. Irrespective of the estimation method, the tube diameter is shown to increase almost linearly with ϕ and increase monotonically with chain-length as we go from a slightly entangled to a more deeply entangled regime. Additionally, it is expected that the tube diameter *a*_pp_ first increases as the entanglement develops but converges if the entanglement structure is fully developed.^74^

### 3.4 Entangled Dynamics

The entangled dynamics of the copolymers is confirmed by a close inspection of the normal mode and dynamic structure factor. The effective relaxation times τ_*p*_^eff^ from the NMA are estimated based on the autocorrelation decay (see Supporting Information for details). The results are shown in Figure 5. The CL250 and CL400 systems (Figure 5a,b) show Rouse-like behavior, indicated by the scaling of τ_*p*_^eff^ ∼ *p* ^–2^. However, as *N* and ϕ increase, the scaling behavior significantly changes with the scaling index close to −3.0, as shown in Figure 5c,d for the CL600 and CL800 systems. Such a change is expected as the development of entanglement asymptotically increases the strength of the topological constraint.^75^

**Figure 5:**
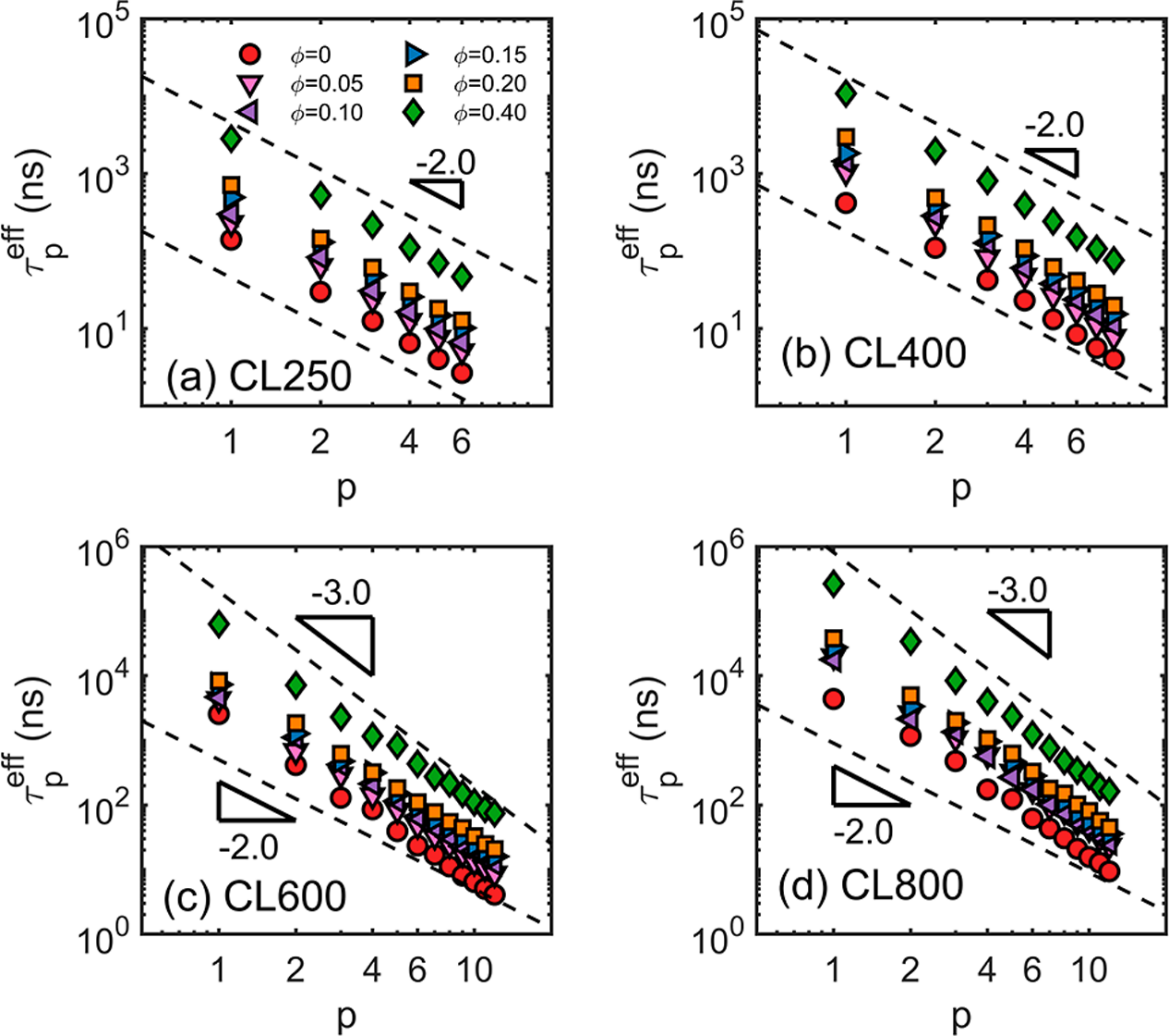
Normal mode analysis to estimate the effective relaxation time τ_*p*_^eff^. The estimated τ_*p*_^eff^ values are plotted as functions of the mode order *p*. For the (a) CL250 and the (b) CL400 systems, the mode-dependence follows the Rouse-like scaling of τ_*p*_^eff^ ∼ *p* ^–2^. However, for the (c) CL600 and the (d) CL800 systems the scaling indexes are close to −3.0, suggesting the entangled dynamics.

Additionally, the DSF analysis (e.g., eq 5) provides another insight into the *q*-specific relaxation processes. The magnitude *S*(*q*,*t*) is calculated with *q* values incrementally ranging from 0.03 to 0.14 Å^–1^. This range of *q* corresponds to physical lengths ranging from 44.9 to 209.4 Å, which span a length scale smaller than the smallest tube diameter (see Figure 4d) to the end-to-end distance $\sqrt{\left\langle R_{ee}^{2}\right\rangle }$ for the longest chains, i.e., the CL800R40 system. The results of the ϕ = 0.0 and ϕ = 0.4 systems are shown in Figure 6 while more results are shown in Figure S12. Overall, all *S*(*q*) curves fully decay to zero, indicating the full relaxation at the respective length scales. At the small length scale (*q* = 0.14), systems with different *N* follow a uniform decaying pattern that can be quantified by the Rouse model (see Figures S13). It is suggested that there is no constraining effect at this length scale. However, at the intermediate and large length scales, the decays deviate from the Rouse-like behavior and are prolonged for the long-chain systems, suggesting the effect of entanglement.

**Figure 6:**
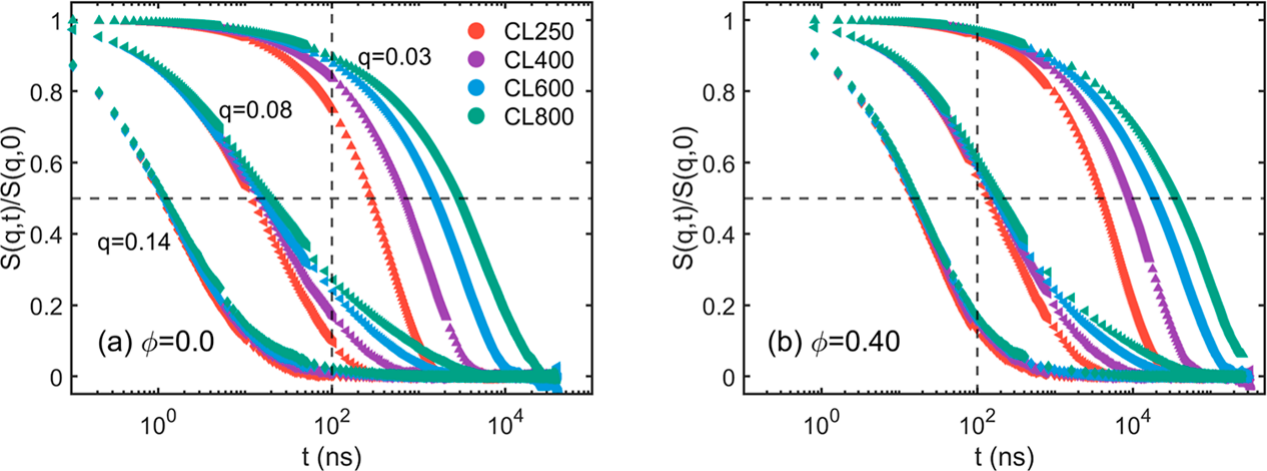
Dynamic structure factor. (a,b) are results for the ϕ = 0.0 and ϕ = 0.4 systems, respectively. As *q* = 0.14 Å^–1^ represents a length scale smaller than the tube diameter, there is no constraining effect such that the decay pattern is independent of the chain length *N*. However, at the intermediate (*q* = 0.08 Å^–1^) and large length scales (*q* = 0.03 Å^–1^), the long-chain systems show prolonged decays while the short-chain systems decay much faster, suggesting that the entanglement effect is stronger for the long-chain systems. The dynamic declaration due to increase in ϕ is indicated by the rightward shift of (b) compared with (a).

The importance of the surviving tube fraction μ­(*t*) and related quantities like Ψ­(*t*) and *P*(*t*) (see Section 2.4) cannot be overstated because they link the conformational evolution of the chains to physical observables. A detailed comparison of the different candidates of μ­(*t*) is provided in Figure 7, including (1) the dark solid line for the prediction by the tube-reptation models by eq 1; (2) the colored dot-dashed line for the LM model prediction by eq 2; (3) the colored dashed line for the tube survival probability Ψ­(*t*) by eq 3 based on the segment survival probability ψ­(*t*) (detailed results are shown in Figures S14–S17); and (4) the colored solid line for the *P*(*t*) decay by eq 4. Please refer to Supporting Information and the original literature for details of the LM model.^17^ In Figure 7, all results are normalized by the respective τ_d_ values, and different colors represent the respective ϕ. The results for different definitions collapse to the respective master curves. The tube-reptation model prediction (dark solid lines) is relatively insensitive to the chain length. It is not surprising that the tube-reptation model predicts prolonged decay because it assumes a strict curvilinear-diffusion mechanism within the tube region, i.e., neglecting CLF and CR effects. Such a model overestimates the constraining strength of the tube. The *P*(*t*) curves most directly probe the dynamics of the linear chains, and the decays are much faster than the predictions of the tube-reptation model. The *P*(*t*) curves completely decay to zero for the CL250 and CL400 systems while there are discernible fluctuations for the CL600 and CL800 systems in the terminal zone. However, the evolution of the disentanglement is quantified, and it is also supported by the tube survival probability Ψ­(*t*). For the CL600 and CL800 systems, the Ψ­(*t*) curves behave quantitatively close to the *P*(*t*) counterparts, although small shoulders remain after *t*/τ_d_ > 0.1. For the CL250 and CL400 systems, the difference between Ψ­(*t*) and *P*(*t*) is discernible through the whole relaxation process, which can be attributed to the fact that the entanglement is not well developed in these cases. It is interesting that Ψ­(*t*) does not seem to decay below a value of ∼0.05 for all systems. For the LM model prediction, the entanglement number from the Z1 analysis is used for the ⟨*Z*⟩ input; the result from the MSD analysis is used for the τ_e_ and τ_d_ inputs. For the CL250 and CL400 systems, it is interesting that the LM model predicts results very close to that for *P*(*t*) in the terminal regime (e.g., *t*/τ_d_ > 1.0). However, for early times the LM model predicts significantly faster decay because of a dominant CLF effect. Such discrepancy is expected because the LM model is parametrized by extensive simulation of the slip-spring model in highly entangled states that are coarser than the CGMD model used in the current work. In fact, the slip-spring model does not distinguish the molecular details of different species while the difference is explicitly modeled in our CGMD simulations. The early-stage discrepancy decreases as the chain length *N* increases. For the CL600 and CL800 systems, the LM model prediction crosses the *P*(*t*) curve at around *t*/τ_d_ = 0.1 and converges back to *P*(*t*) at around *t*/τ_d_ = 1.0, indicating that the LM model describes the overall dynamics of the entangled systems in a reasonably quantitative manner. It is also interesting that the DSF decays can also be normalized by the system-specific τ_d_ as shown in Figure S18. The fact that μ­(*t*), *P*(*t*), Ψ­(*t*), and *S*(*t*) can be well-normalized by τ_d_, which itself follows the ∼10^2.7ϕ^ scaling, suggests the homogeneous relaxation at the chain-level. The homogeneous relaxation is controlled only by ϕ, as the monomeric friction increases with ϕ.

**Figure 7:**
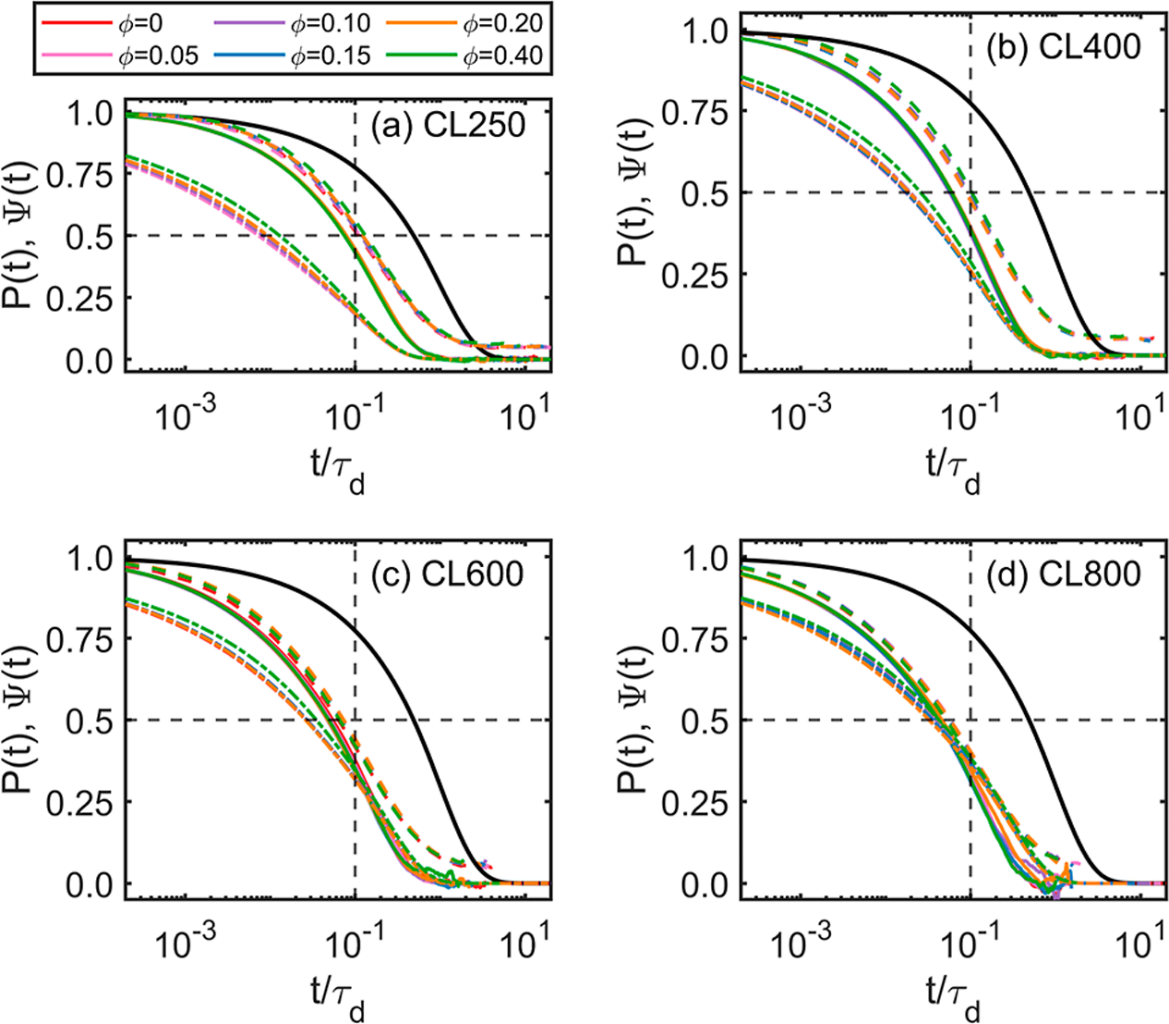
Molecular relaxation of the entangled systems. All results are normalized by the system-specific τ_d_. Different colors represent the respective ϕ. The dark solid lines are the predictions of the reptation model (eq 1); the dot-dashed lines are the predictions of the Likhtman–McLeish model (eq 2); the dashed lines are the tube survival probability Ψ­(*t*) (eq 3); the solid lines are the autocorrelation of the end-to-end vector *P*(*t*) (eq 4). The reptation model predicts the slowest dynamics because it overestimates the entanglement constraint. For the (a) CL250 and (b) CL400 systems, the LM model prediction matches with *P*(*t*) better than Ψ­(*t*) in the terminal regime; on the other hand, for the (c) CL600 and (d) CL800 systems, Ψ­(*t*) matches with *P*(*t*) better in all regimes.

### 3.5 Bulk Viscoelasticity

The relaxation modulus *G*(*t*) represents the bulk viscoelastic property and reflects the evolution of μ­(*t*). See Supporting Information for detailed calculation. Figure 8 compares the MD results for *G*(*t*) (solid lines) with the predictions of two different models, i.e., the LM model (dot-dashed lines) and the double-reptation model (dashed lines). The later model predicts a μ­(*t*) ∼ *P* ^2^(*t*) relation^76,77^ and effectively quantifies the CLF effect with the dilation of the constraining tube. A recent simulation study has shown that the double-reptation model works well for interpreting the MD results of the Kremer-Grest model.^78^ For slightly entangled systems, as shown in Figure 8a, the MD results fall between the double-reptation and LM model predictions. The LM model includes the CLF effect, leading to faster relaxation, while the double-reptation model overestimates the strength of the tube constraint, leading to slower relaxation. It should be noted that the double-reptation model results in a less restricted dynamic behavior than the original tube model.^61^ Therefore, the dynamics of the original reptation model (not shown) is expected to be even slower than the dynamics of the double-reptation model. Overall, the predictions of the double-reptation model are more accurate for the short-chain cases. Although the plateau of the relaxation modulus is not fully developed, its time-dependent behavior qualitatively agrees with the conceptual picture of entangled dynamics. On the other hand, the plateau of the relaxation modulus is fully developed for the well-entangled systems such that *G*(*t*) does not decay significantly until *t* > 10^3^ ns. The results of the CL800 systems are shown in Figure 8b, where the horizontal dashed line is the experimental value of the plateau modulus, *G*_e_ = 0.2 MPa, for PDMS at 413 K.^70^ During the early stage of relaxation, i.e., *t* < τ_e_, both predictions of the double-reptation and the LM models agree quantitatively with the MD results, and the difference between the two models are negligible because both models include the CLF effect. However, the double-reptation model overpredicts the constraint strength and causes the elevated plateau modulus in the later regimes of relaxation *t* > τ_e_. As the molar ratio ϕ increases, the prediction of the LM model agrees markedly well with the MD result. The numerical fluctuation at the end of the *G*(*t*) curve is expected due to the challenge of autocorrelation function calculation, especially for the virial stress tensor used to sample the *G*(*t*) curve.^53^ The *G*(*t*) curves for the CL400 and CL600 systems are shown in Figure S19. The LM model predicts the *G*(*t*) behavior the best for the well-entangled system due to its slip-spring essence, despite the discrepancy of the *P*(*t*) early decay shown in Figure 7.

**Figure 8:**
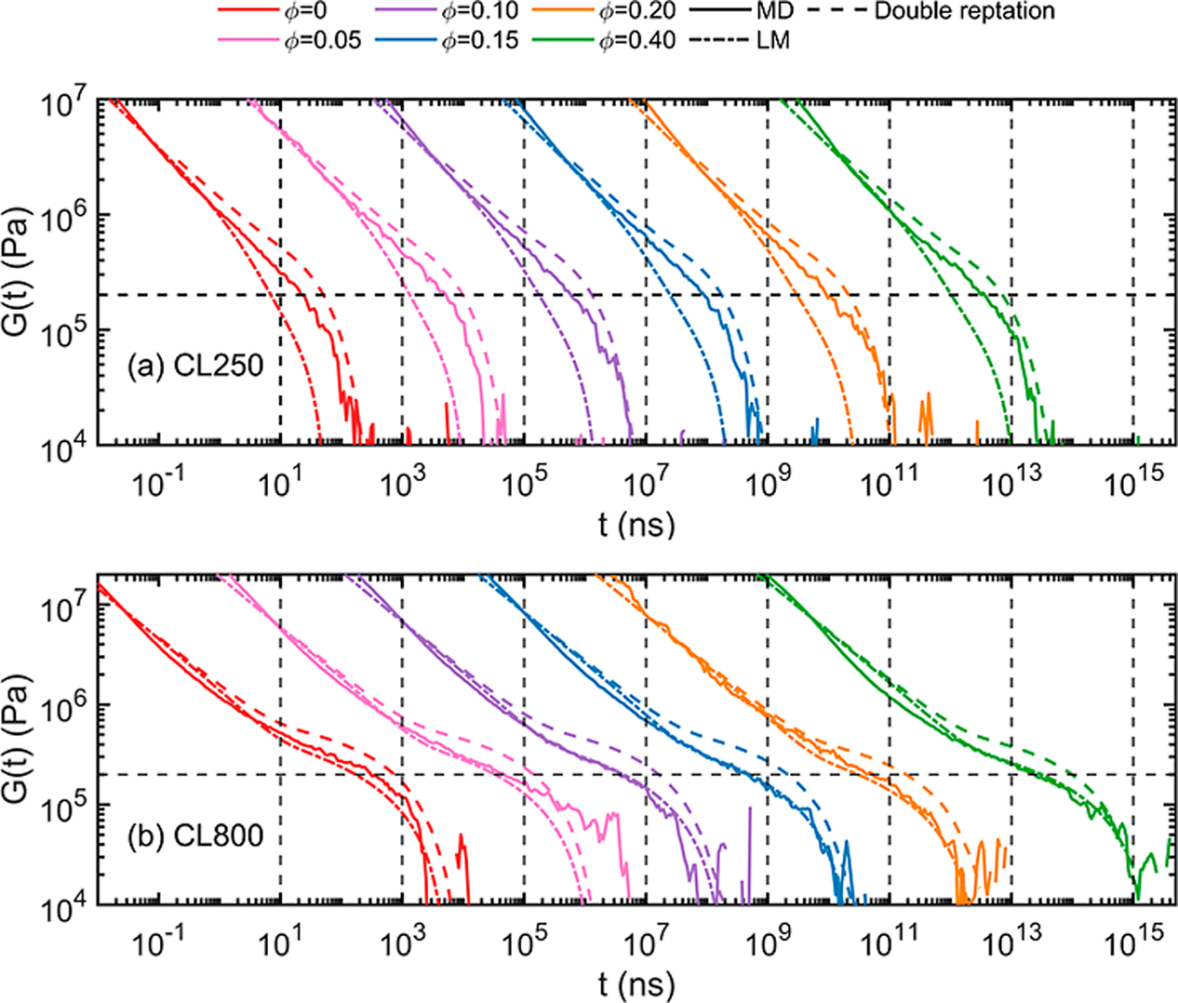
Relaxation modulus. The solid lines are the MD simulation results. The dashed and the dot-dashed lines are the predictions of the double-reptation model and of the Likhtman–McLeish model (see eq S1 in Supporting Information). Different colors represent the respective ϕ. Curves are horizontally shifted by 2 orders for visual clarity. (a) In the case of slightly entangled system (*N* = 250), the double-reptation model quantifies the decay of *G*(*t*) better. (b) On the other hand, the LM model describes the decay of *G*(*t*) better for the well-entangled system (*N* = 800), especially in the plateau and terminal regimes where the entangled dynamics dominates the decay.

### 3.6 Comparison with Experimental Results

The comparison of the viscosity results are shown in Table 1 and Figure 9a. The converted dynamic viscosity η_d_ derived from the simulation-based *G*(*t*) curves (according to the LM model prediction with parameters from CGMD) quantitatively matches with the experimental measurement. See Supporting Information for detailed calculation. Although the discrepancy between the simulation-based and experimental viscosity is not negligible, it is attributed to the fact that copolymer liquids may have impurities, additives, and polydispersity. However, these factors are not considered at all in the CGMD simulations and viscosity derivations. It should be emphasized that no additional fitting is involved for deriving the simulation-based results. It is suggested that the molecular relaxation at the chain level is indeed homogeneous that can be quantified by the LM model. It is also suggested that our CGMD model captures well the ϕ-dependent structural and dynamic properties. Additionally, the experimental and simulation-based relaxation modulus of the two copolymer gums are shown in Figure 9b,c for a direct comparison. The dot-dashed curves are experimental results converted from the rheological complex modulus G*­(ω). The dark solid curves are the LM model predictions with chain length *N* that correspond to the number-averaged molecular weights *M*_n_. In Figure 9b,c, the LM model predictions with other chain lengths *N* are represented by the colored curves. It is worth noting that the LM model predicts a plateau modulus that matches the experimental results, suggesting that the CGMD simulation is indeed powerful for sampling the structural and dynamic properties of the copolymer systems. However, the LM model predictions of the *G*(*t*) curves slightly deviate from the experimental curves at the early stages of the relaxation (*t* < 10^1^ s). Note that the experimental complex modulus G*­(ω) curves were constructed by TTS, which may be affected by the fact that the reference temperature *T*_0_ = −173.0 K is close to the glass transition temperature of the copolymers (LVM and SE). The low-temperature/high-frequency regime may not accurately reflect the segmental dynamics of the copolymer. The discrepancy in the early stage is also attributed to the fact that the LM model only considers monodisperse and entangled systems. However, the experimental systems are polydisperse (as suggested by *M*_w_/*M*_n_ in Table 2) and very likely have unentangled chains. The influence of the polydispersity is also indicated by the different terminal behaviors of the simulation-based and experimental *G*(*t*) curves (*t* > 10^2^ s). We hypothesize that the combination of the simulation-based results (with different *N*) can possibly quantify how the polydispersity affects the experimental behaviors of the copolymer systems. The molecular relaxation of LVM (ϕ = 0.035) is faster than that of SE (ϕ = 0.053) since their monomeric frictions and molecular weights are different, an observation that is consistent with our previous study.^41^ Additionally, the entanglement modulus is 0.22 MPa for the simulation-based prediction while the experimental value is 0.20 MPa.^70^ Overall, the viscoelasticity of the copolymer liquids and gum are quantitatively captured by the simulation-based prediction.

**Figure 9:**
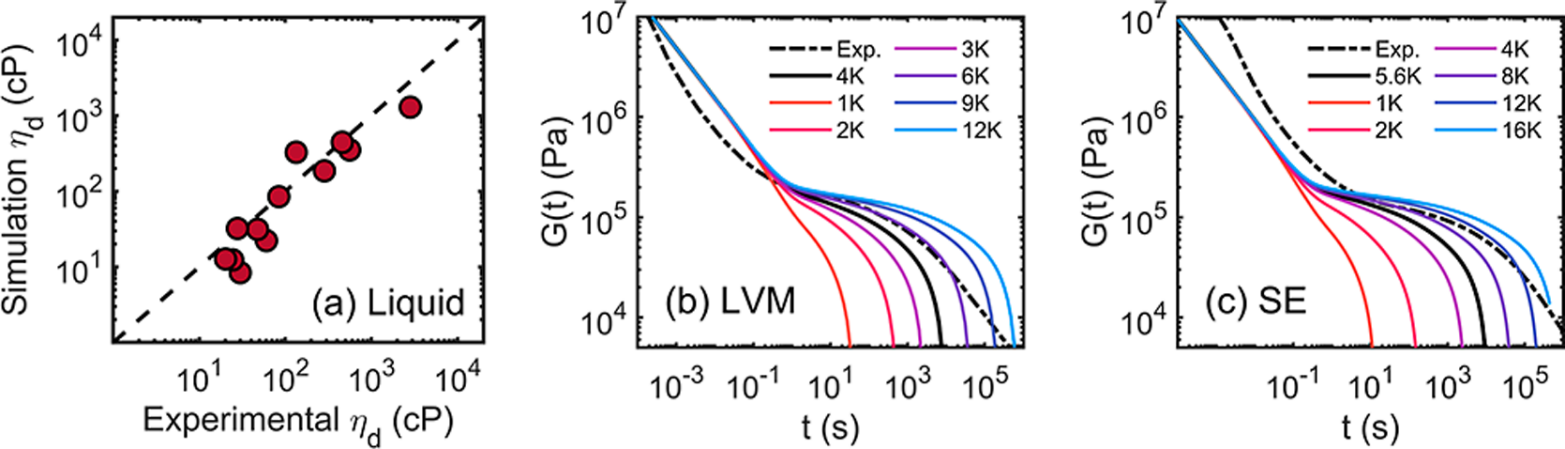
Experimental characterization of the viscoelasticity of the copolymer liquids and gums. (a) Comparison of the dynamic viscosity η_d_ from experimental measurement and simulation-based prediction. (b,c) are the relaxation moduli derived from experimental complex modulus G*­(ω) and from simulation-based LM model prediction. The dashed lines are for *G*(*t*) that matches *M*_n_ of the materials while the colored lines are for quantifying the chain-length effect on *G*(*t*).

### 3.7 Local Structural and Dynamic Properties

In this section, we explore the connection between the local copolymer composition (ϕ_loc_) and the local structural properties. It starts with the time-averaging operation on the MD trajectories according to eq 9. Figure 10a is an example of the average trajectory of a chain from the CL800R40 system. The average trajectory is termed mean path henceforth. The mean path is smoother than the instantaneous trajectory, and the local fluctuation of the mean path is significantly suppressed. The primitive path of the same chain identified by the Z1 algorithm is provided in Figure 10b for comparison. In fact, the averaging operation was a common practice before the development of more quantitative methods, like the PPA analysis and the Z1 algorithm.^54,79,80^ It is worth noting that (1) the mean path preserves more local fluctuation than the primitive path, as the Z1 algorithm optimizes geometrically the single-chain conformation into straight segments and (2) the deviation of the mean path from the instantaneous trajectory is more notable at the chain ends, while the Z1 primitive path has the ends fixed in space. Additionally, a longitudinal vector can be defined by ***r***_*i*+2_–***r***_*i*–2_ (and normalized by its length) for a bead ***r***_*i*_. It is easy to define two other auxiliary unit vectors that are mutually perpendicular to each other and to the longitudinal vector. The three vectors form a local orthogonal coordinate system, based on which the local displacement amplitudes, *r*_⊥_ and *r*_∥_, are calculated. The first and the last 2% of the beads are neglected to avoid chain-end effects. Figure 10c,d plots the results of *r*_⊥_ and *r*_∥_ (normalized by the ensemble average) as functions of the bead’s fractional location (*s*) within a probe chain that is chosen randomly from the CL800R40 system. For comparison, these plots are overlaid with the corresponding plots for the local composition (more precisely 1 – ϕ_loc_). Figure 10c shows that both curves strongly track with each other with an overall fluctuation of ±20%. In other words, the transverse displacement *r*_⊥_ is usually high when ϕ_loc_ is low and vice versa. On the other hand, the correlation between longitudinal bead displacement *r*_∥_ and ϕ_loc_ appears weak, as Figure 10d shows.

**Figure 10:**
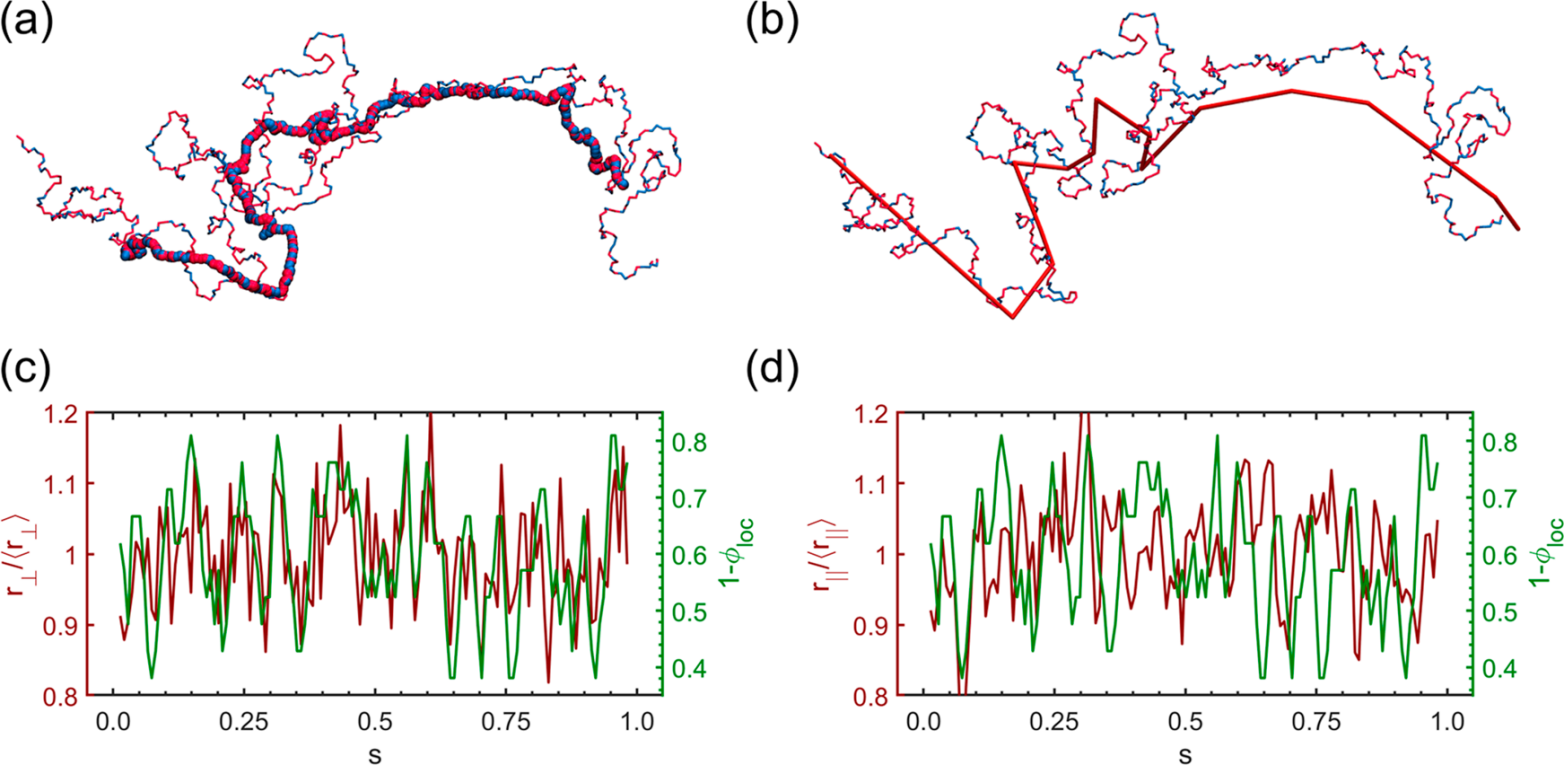
Mean path and the correlation between the local displacement and the local composition. (a) Mean path according to eq 9. (b) Primitive path generated by the Z1 algorithm for a molecule selected from the CL800R40 system. (c,d) are the local transverse displacement *r*_⊥_ and longitudinal displacement *r*_∥_ plotted against the local composition ϕ_loc_. The *r*_⊥_ results show a stronger correlation with the local composition than the *r*_∥_ results.

Results of ⟨*r*⟩ are plotted as functions of the bulk ϕ as shown in Figure 11a. The time window for the averaging operation is τ_ave_ = τ_e_(ϕ = 0.0). Note that the composition has no variation at all for the ϕ = 0.0 case. It is shown that ⟨*r*_⊥_⟩ is larger than ⟨*r*_∥_⟩. Interestingly, the local displacements decrease monotonically as ϕ increases, implying the slowdown in the relaxation dynamics as previously discussed. The ϕ-dependence shown in Figure 11a is insensitive to the chain length *N*, suggesting that ⟨*r*_⊥_⟩ and ⟨*r*_∥_⟩ are indeed local properties, which is already seen in the DSF results of large *q* values that are related to small length scales. Additionally, a similar analysis is conducted with τ_ave_ equal to the system-specific τ_e_, and the results are shown in Figure 11b. The local displacements are monotonically increasing functions, in contrast to the results shown in Figure 11a. Such an observation corresponds to the fact that the tube diameter *a*_pp_ increases with ϕ, as shown in Figure 4d. It is noteworthy that different batches of equilibrium MD trajectory are used for Figure 11a,b, so that there are small differences for the ϕ = 0.0 systems. There are one hundred configurations in a batch of mean paths. It is an intentional choice to prove that the time-average operation is robust and that the presented results are statistically meaningful.

**Figure 11:**
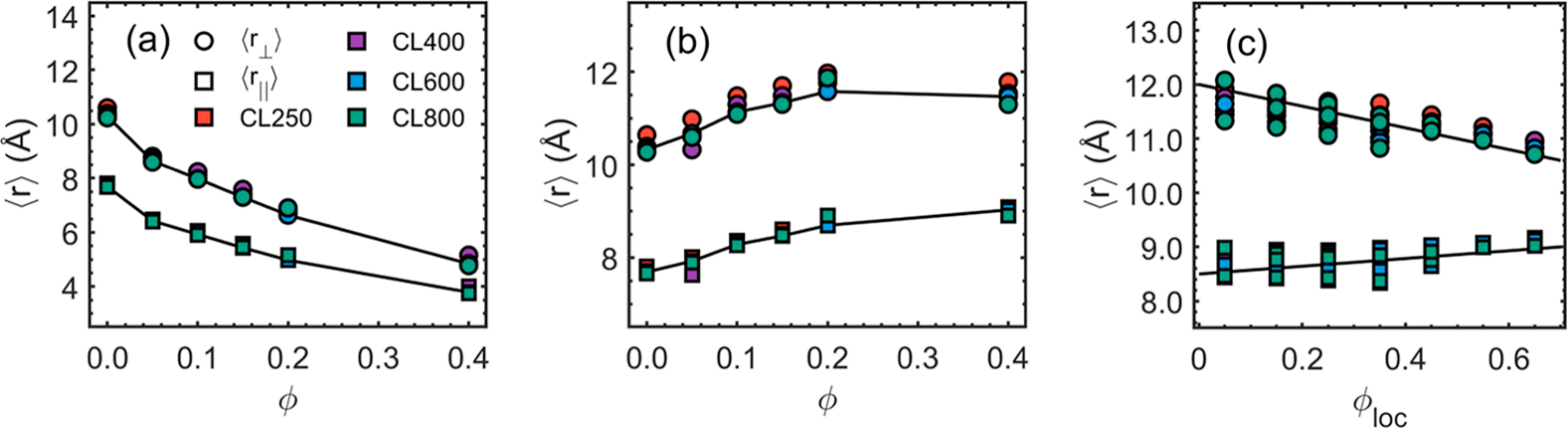
Local structural properties by analyzing the mean path. (a) Average local displacement ⟨*r*⟩ with the time window τ_ave_ = τ_e_(ϕ = 0.0) fixed. (b) Average local displacement ⟨*r*⟩ with the time window τ_ave_ equal to system-specific τ_e_. (c) Local displacement ⟨*r*⟩ for all systems as functions of ϕ_loc_ with the time window τ_ave_ equal to system-specific τ_e_. The line is a guide to the eye.

Markedly, a dependency of ⟨*r*_⊥_⟩ on the local composition ϕ_loc_ exists, as shown in Figure 11c, regardless of the chain length and the bulk composition ϕ, suggesting that ⟨*r*⟩ is a local property that is also sensitive to ϕ_loc_. The system-specific τ_e_ was used for τ_ave_ to derive the results shown in Figure 11c. Interestingly, a negative ϕ_loc_-dependence is observed for ⟨*r*_⊥_⟩, which is qualitatively close to the results in Figure 11a (with τ_ave_ = τ_e_(ϕ = 0.0) used). However, ⟨*r*_⊥_⟩ decreases with ϕ_loc_ less significantly than its decrease with ϕ, implying that the slowdown at the segment-level is weaker than the slowdown shown at the chain-level. In Figure 11c, the results of ⟨*r*_∥_⟩ seem to be independent of the local composition ϕ_loc_. It is noteworthy that the implication regarding the slowdown in the relaxation is only qualitative in the sense that the mean path and the metrics that ⟨*r*_⊥_⟩ are ⟨*r*_∥_⟩ are not explicitly defined as functions of time, in contrast to other works that treat the metrics in a time-explicit fashion.^9,81^ Nevertheless, the metrics reveal how the local structural and dynamic properties are related to ϕ and ϕ_loc_.

To provide a deeper insight into the segmental relaxation, the analyses of MSD, NMA, and DSF are performed with the local composition ϕ_loc_ explicitly differentiated. The CL800R40 system is used as an example because it is the most entangled and has the most variation in ϕ_loc_. The *g*_1_ results are shown in Figure 12a. It is shown that the different ϕ_loc_ “species” qualitatively follow the bulk-average behavior of the MSD results shown in Figure 2c,d. However, they quantitatively differ from each other at the early stage (i.e., prior to τ_e_). The peak times τ_peak_ are plotted as a function of ϕ_loc_ in Figure 12b. The peak of the rescaled MSD decreases and shifts rightward as ϕ_loc_ increases, showing the segmental dynamics is decelerated. At longer time scales, the different species relax in a more collective way, suggested by the fact that all curves converge closer to each other beyond τ_e_. It seems that the curves start to deviate from each other after τ_R_ but the divergence is attributed to the insufficient statistic for the long-time autocorrelation calculation.

**Figure 12:**
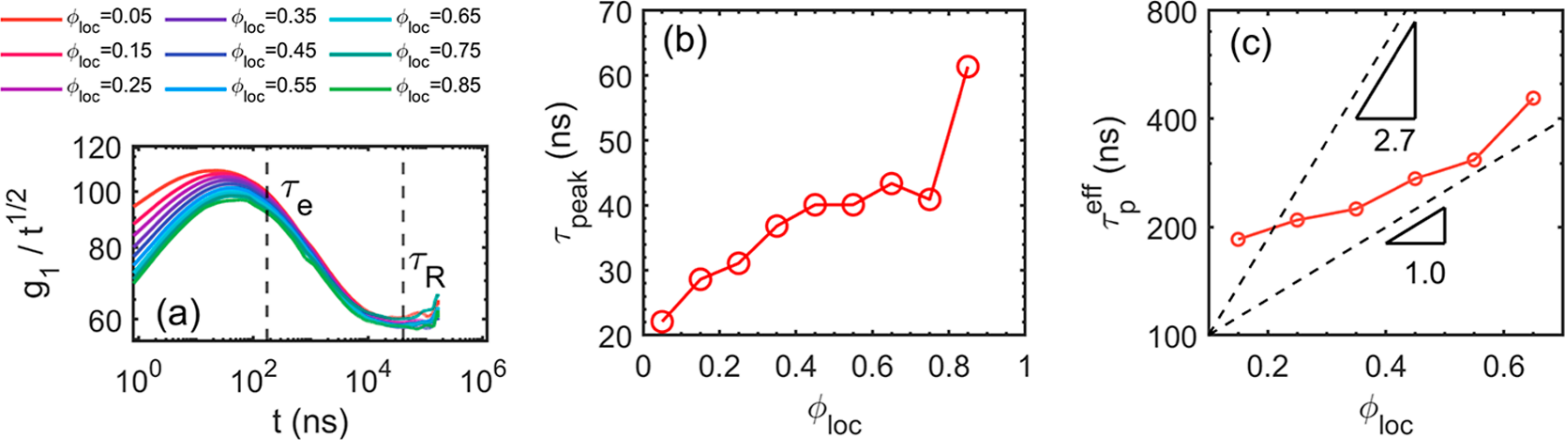
Dependence of the local dynamics on the local composition ϕ_loc_. (a) Rescaled *g*_1_ result. (b) Peak times of the rescaled MSD curves as a function of ϕ_loc_. (c) Estimated effective relaxation time τ_*p*_^eff^ with a scaling different than the bulk behavior.

As the *g*_1_ results focus on the dynamics of individual beads, the normal mode (i.e., eq 4) is applied to quantify the collective dynamics of the internal segments that include 51 beads whose size is below the entanglement threshold. A 51-bead segment is slightly smaller than an entanglement segment. Not all species are processed due to the statistical limitation, but the successfully identified values of the effective relaxation time, τ_*p*_^eff^, are plotted in Figure 12c. The monotonic increase of τ_*p*_^eff^ as a function of ϕ_loc_ indicates the ϕ_loc_-dependent dynamics. However, the dependence of τ^eff^ ∼ ϕ_loc_ shows a weaker correlation than the bulk behavior (i.e., τ^eff^ ∼ 10^2.7ϕ^). The incoherent dynamic structure factors are also calculated. The results are plotted in Figure S20, showing that the segmental relaxation is decelerated as ϕ_loc_ increases, while the relaxations at larger length scales are collective. However, the difference of the incoherent DSF decays is minor, as the characteristic times only increase slightly with ϕ_loc_. The analyses of MSD, NMA, and DSF suggest that the segmental relaxation is heterogeneous. However, the heterogeneity manifests at the length scale that is smaller than an entanglement segment, suggesting the reason why the molecular relaxation of the copolymers is collective and homogeneous at the chain level.

### 3.8 Discussion

A chemistry-specific CGMD model is used to study the PDMS-*co*-PDPS random copolymers in this work. Unlike generic models like the Kremer–Grest model relying on parameter-mapping,^82^ our work demonstrates with chemical details that the accumulation of the ϕ_loc_-dependence at small length scales fundamentally determines the collective ϕ-dependence of the bulk. Additionally, the entanglement length is shown to be the threshold length scale relevant to the self-concentration effect. This observation contrasts with the PDMS-based block copolymers.^83^ For the block copolymer, scales of the different blocks are usually well-separated and thus chosen as the threshold. For the random copolymers, a theory that quantitatively bridges the local (ϕ_loc_-dependent) heterogeneity and the bulk (ϕ-dependent) homogeneous dynamics is in a premature state because of the challenge to quantitatively account for the statistical sequence. Thermodynamically consistent models based on mean-field theory and other advancements of multiscale theories are needed to improve the understanding of the local-to-bulk correlation.^31,84^ Random block copolymers represent a unique model system to study the local-to-bulk correlation. For example, Steinmuller et al. studied the mechanical properties of the model random block copolymers.^85^ It was found that the microscopic separation of the random block copolymers affects the entanglement structure but not the viscoelasticity. On the other hand, the heterogeneous segmental relaxation causes the emergence of a shoulder in the relaxation modulus *G*(*t*). Additionally, knowledge of binary compatible polymer blends may help to understand the local-to-bulk correlation. For example, fully miscible blends of polystyrene with its oligomers showed strong dynamics heterogeneity in the segmental dynamics, which can only be qualitatively described by the Lodge–McLeish model since there is no compositional fluctuations involved.^86^ Incorporation of all these additional factors, like entanglement, statistical sequence, and compatibilizer, can deepen our understanding of the mechanical properties of the copolymers or blends, fostering opportunities for novel material design.^87–89^

## 4 Conclusion

In this study, we investigate the entangled dynamics and bulk viscoelasticity of slightly and well entangled PDMS-*co*-PDPS copolymer in melt density via long-term CGMD simulations. The molar ratio of the diphenyl component ϕ and the chain length of the linear molecule *N* are systematically varied such that the model systems cover up to ten entanglements per chain. We closely examine the structural and dynamic properties of the model system. The entanglement analysis shows the development of entanglement structure as ϕ and *N* increase. Although the number of entanglements varies weakly with the bulk copolymer composition ϕ, the overall chain stiffness increases with ϕ, which leads to a monotonically increasing tube-diameter and primitive path length as a function of ϕ. The model copolymers are fully miscible because the CGMD simulations are conducted at 550.0 K, a sufficiently high temperature that prevents microphase segregation. The analysis of local structure and dynamics (dependent on ϕ_loc_) suggests that the heterogeneity is limited to the entanglement length. It is shown that the entangled dynamics is controlled by the monomeric friction which is strongly correlated with ϕ but less sensitive to ϕ_loc_. Therefore, the entangled dynamics of the copolymer system is collective and homogeneous at the chain level and can be well quantified by the Likhtman–McLeish model. Particularly, the entangled dynamics is fully revealed and systematically mapped by the relaxation modulus *G*(*t*). The clear connection between ϕ, *N*, and *G*(*t*) of the copolymers enables quantitative prediction of the macroscopic viscoelasticity of the materials. It is also shown that the simulation-based prediction agrees with experimental viscosity and relaxation modulus. Although it is expected that the relaxations at the chain- and segment-levels are, respectively, homogeneous and heterogeneous, the current work represents a unique effort to confirm and apply such physical ansatz to the model PDMS-*co*-PDPS random copolymer. The protocol of predicting experimental viscoelasticity via CGMD simulation is valuable in understanding and designing the copolymer materials for scientific and industrial applications.

## Supplementary Material



## Notes

Details of the CGMD simulation: time rescaling factors; MSID; characteristic times; NMA; coherent and incoherent dynamic structure factors; segment survival probability; relaxation modulus; simulation-based viscoelasticity; experimental details: viscosity of copolymer liquids; and complex modulus of copolymer gums (PDF)
