# Supplementary material for: Understanding Viscoelasticity of an Entangled Silicone Copolymer via Coarse-Grained Molecular Dynamics Simulations
Source: Macromolecules. 2025 Aug 11;58(16):8943–57. doi: 10.1021/acs.macromol.5c01192 (PMC12503380; doi:10.1021/acs.macromol.5c01192)
Supplement: Supplementary file 1 [file ma5c01192_si_001.pdf]

Supporting Information to

Understanding Viscoelasticity of an Entangled Silicone Copolymer via Coarse-Grained Molecular Dynamics Simulations

*Weikang Xian,[1] Amitesh Maiti,[2] Andrew P. Saab,[2] and Ying Li[1,]**

[1] Department of Mechanical Engineering, University of Wisconsin-Madison, Madison, Wisconsin 53706-1572, United States

[2] Lawrence Livermore National Laboratory, Livermore, California 94550, United States

E-mail: yli2562@wisc.edu

Table S1. Time rescaling factor S_τ that maps the dynamics of the coarse-grained molecular dynamics simulation back to the physical relevant scales. $S_\tau = D_{\mathrm{CG}}/D_{\mathrm{AA}}$, where D_{CG} and D_{AA} are the diffusion coefficients from the coarse-grained and all-atomistic simulations, respectively.[1]

$\boldsymbol{\phi}$	0.0	0.05	0.1	0.15	0.2	0.4
$\boldsymbol{S_\tau}$	2.86	5.36	6.98	8.89	10.17	22.31

Mean-squared internal distance. To confirm the equilibrium configuration of the model system, the mean-squared internal distance (MSID) is calculated, and the results are shown in **Figure S1**. The development of the plateau of the MSID curves, as the internal segment length increases, indicates the full equilibrium state for a model system. Additionally, the last points of the MSID curves are used to calculate the mean-squared end-to-end distance (MSD), $\langle R_{\mathrm{ee}}^2 \rangle$, for the corresponding systems. MSID quantifies the size of a partial segment that internally belongs to a linear chain molecule, $\langle R^2(s) \rangle$, where R is the distance between the head and tail of the partial segment, and s ranges from 1 to N. Note that $\langle R^2(s) \rangle = \langle R_{\mathrm{ee}}^2 \rangle$ when $s = N$.

Viscosity of copolymer liquids. 9 types of the PDMS-*co*-PDMS linear random copolymers and 3 pure PDMS homopolymers were purchased from Gelest. **Table 1** in the main text lists detailed material information. Brookfield rotational viscometer was used to measure the steady-state dynamic viscosity η_{d} of the copolymer liquids. The materials were used as received. The strain-rates of measurement varied from 3.0 to 20.0 s^{-1}, within the linear viscoelastic regime. At each temperature, viscosity results with at least five different strain-rates were averaged. The measurement temperature changed from 298.0 to 373.0 K. Arrhenius-like relation was used to extrapolated results of viscosity at 550.0 K. the temperature-dependent results of η_{d} were fitted against $\log(\eta_{\mathrm{d}}) = aT^{-1} + b$ where a and b are fitting parameters. The fitting and extrapolation are shown in **Figures S2** to **S5**. Note that the nominal η_{d} values shown in **Figures S2** to **S5** were

calculated based on the kinematic viscosity η_{k} provided by Gelest, assuming the copolymer liquids have the same density and pure PDMS (0.97 g/cm^3). The η_{d} results at 550.0 K are summarized in **Table 1** in the main text. 1 cP equals to 0.001 Pa·s.

Complex modulus of copolymer gums. The complex modulus $G^*(\omega)$ of two highly entangled copolymer gums are from our previous study.[2] The results is shown in **Figure S6** with the original master curves and the fitting of the generalized Maxwell model with 20 modes. Time-temperature superposition (TTS) was applied to construct the master curves. The reference temperature was $T_0 = -173.0$ K. The shift factor a_{T} in the TTS adopted the WLF function form, as given by equation S1, where C_1 and C_2 are fitting parameters. $C_1 = 7.9$ and $C_2 = 41.0$ K for LVM. $C_1 = 7.2$ and $C_2 = 31.3$ K for SE. The experimental relaxation modules is converted by $G(t) = \sum_i g_i \exp(-t/\tau_i)$, where g_i and τ_i are the fitting parameters of the generalized Maxwell model.

$$\log a_{\mathrm{T}} = -\frac{C_1(T-T_0)}{C_2+T-T_0} \qquad \text{eq. S1}$$

Mean-squared displacement. To confirm the full relaxation of the model systems, the mean-squared displacement (MSD), g_1, g_2, and g_3 (see the definition in the main text) are calculated. The results are shown in **Figures S7** to **S10** for the CL250 to CL800 systems, respectively. In the figures, the marked horizontal and vertical dashed lines are for the corresponding $\langle R_{\mathrm{ee}}^2 \rangle$ values and estimations of the disentanglement time τ_{d}. For the systems whose g_3 curves do not approach the corresponding $\langle R_{\mathrm{ee}}^2 \rangle$, linear extrapolation is used to estimate τ_{d} by assuming $g_3 \sim t$.

Characteristic times. The characteristic times τ_{e}, τ_{R}, and τ_{d} identified from the MSD results are shown in **Figure S11**. The are used to calculate the monomeric relaxation time τ_0 according to

three conceptually equivalent expressions: (1) $\tau_0 = \tau_{\mathrm{e}} N_{\mathrm{e}}^{-2}$; (2) $\tau_0 = \tau_{\mathrm{R}} N^{-2}$; and (3) $\tau_0 = \tau_{\mathrm{d}} N_{\mathrm{e}} N^{-3}$. The estimation of τ_0 is shown in **Figure 3** of the main text.

Normal mode analysis. The relaxation of the copolymer is further examined by the decoupled relaxation time, τ_p, associated with the orthonormal coordinates. While the Rouse or the tube-reptation model predict the relation of $C_p(t) = \exp(-t/\tau_p)$ for each mode, a refinement with a stretched exponential, $C_p(t) = \exp\left[-(t/\tau_p^*)^{\beta_p}\right]$, is more often used to quantify MD result.[3] The stretched exponential yields the effective relaxation time $\tau_p^{\mathrm{eff}} = \tau_p^*/\beta_p \Gamma(\beta_p^{-1})$, where Γ is the gamma function. The characteristic times τ_p^{eff} estimated by the normal mode analysis are plotted as functions of the bulk molar ratio ϕ of the diphenyl component in **Figure S11**. The results plotted as functions of the mode order p are shown in **Figure 5** in the main text. It is shown that the scaling of $\tau_p^{\mathrm{eff}} \sim 10^{2.7\phi}$ is reproduced and consistent with our previous works that study the copolymer with all-atomistic and unentangled coarse-grained molecular dynamics simulations.[1,4] The change of the p-dependent scaling behavior indicates the stronger entanglement effect as N increases.

Coherent dynamic structure factor. To quantify the relaxation of the chain molecules at different length scales, the coherent dynamic structure factor (DSF) is calculated according to equation 7 in the main text. The results of the $\phi = 0.0$ and $\phi = 0.4$ systems are shown in **Figure 6** in the main text while the results of other systems are shown in **Figures S12**. In **Figures S13**, the DSF results of CL600 systems are plotted together with the Rouse model prediction. For the Rouse model prediction, no fitting is involved, and it is calculated according to the following equation:[5]

$$S(q,t)=\frac{1}{N}\exp\left\{-\frac{q^2}{6}g_3(t)\right\}\left\{\sum_{i=1}^{N}\sum_{j=1}^{N}\exp\left[-\frac{2q^2}{3}\sum_{p=1}^{N-1}\langle \boldsymbol{X}_p^2\rangle\left(\left[A_{pi}-A_{pj}\right]^2 + 2A_{pi}A_{pj}\left[1-C_p(t)\right]\right)\right]\right\} \quad \text{eq. S2}$$

where $A_{pi}=\cos\frac{\pi p}{N}\left(j-\frac{1}{2}\right)$ and $C_p(t)=\exp\left[-(t/\tau_p^*)^{\beta_p}\right]$. The DSF decays are well quantified by the Rouse model at large q but deviate at small q, indicating the entanglement effect at small q (large length scale). In **Figure S18**, the DSF curves are rescaled by the corresponding t_d. The collapsing of the curves into respective master curves with different ϕ values suggests that the entangled dynamics is universal to all systems regardless of ϕ**.** More importantly, the molecular relaxation is homogeneous at the chain-level.

Segment survival probability. The primitive path is obtained from the Z1 analysis.[6,7] With the identified primitive path, the segment survival probability $\psi(s,t)$ (see definition in the main text) is then calculated. The results are shown in **Figures S14** to **S17** for the CL250 to CL800 systems, respectively. All segment survival probability curves decay to a plateau value 0.05 as they approach full relaxation. The tube survival probability function $\Psi(t)$ is then calculated by the corresponding segment survival probability (see equation 3 in the main text).

Relaxation modulus. The relaxation modulus for the CL400 and CL600 systems are shown in **Figure S19**. The solid, dashed, and dot-dashed lines are for the MD calculation, the double-reptation model prediction,[8–12] and the Likhtman-McLeish (LM) model prediction,[13] respectively. As the level of entanglement develops, due to the increase in the chain length and molar ratio ϕ, the MD results are quantified by the Likhtman-McLeish model better than the double-tube model. The MD results are calculated by the on-the-fly multi-tau algorithm (see the main text).[14] For the

Likhtman-McLeish model, the relaxation modulus $G(t)$ is calculated according to the following equation:

$$G(t) = G_{\mathrm{e}}\left[\frac{4}{5}\mu(t)R(t) + \frac{1}{5Z}\sum_{p=1}^{Z-1}\exp\left(-\frac{p^2 t}{\tau_{\mathrm{R}}}\right) + \frac{1}{Z}\sum_{p=Z}^{N}\exp\left(-\frac{2p^2 t}{\tau_{\mathrm{R}}}\right)\right] \qquad \text{eq. S3}$$

where G_{e} is the entanglement plateau modulus $G_{\mathrm{e}} = \rho k_{\mathrm{B}}T/N_{\mathrm{e}}$. ρ is the number-density of CG beads. N_{e} is the length of entanglement. The first term describes the combination of the tube-reptation-like escape. The second term quantifies the longitudinal modes relaxation (segment reorientation due to the contour length fluctuation effect). The third term quantifies the fast Rouse motion inside the tube. $\mu(t)$ is the surviving tube fraction of the LM model, as given by equation 2 in the main text. The Z value is the entanglement number estimated from the Z1 analysis. The Rouse time τ_{R} and entanglement time τ_{e} are estimated from the MSD curves. $R(t)$ is the function to account the constraint release (CR) effect, and it is calculated according to the following equation:

$$R(t) = 1 - \frac{1.8}{Z}\left(\frac{t}{t_{\mathrm{e}}}\right)^{1/4} \qquad \text{eq. S4}$$

For the double reptation model, the $\mu(t)R(t)$ is replaced by $P^2(t)$ with $P(t)$ the autocorrelation of the end-to-end vector.

Simulation-based viscoelasticity. The simulation-based relaxation modulus $G(t)$ is generated according to the LM model to predict the viscoelasticity of the copolymers. Extrapolation of structural and dynamic properties is needed for the experimental systems, since the chain length N and mean-ratio of the diphenyl monomer ϕ of the CGMD simulation systems may not exactly match those parameters of the experimental systems. Specifically, the entanglement number Z is calculated by $Z = N/N_{\mathrm{e}}$ with N_{e} and N as the entanglement length from the CL800 simulation

systems and the chain length of the target system. Additionally, the disentanglement time t_{d} is extrapolated by assuming $t_{\mathrm{d}} \sim N^{3.4}$ and the Rouse time t_{R} is extrapolated by assuming $t_{\mathrm{R}} \sim N^{2.0}$. The extrapolations are based on the CGMD results sampled from the CL800 simulation systems. The CGMD results of t_{e} and density from the CL800 systems are used without modification. The changes of structural properties of the entanglement are small, according to **Figure 4** in the main text. Therefore, structural properties estimated from the CL800R5 simulation are used for the PDV03 liquids, LVE and SE gums. Structural properties of the CL800R15 simulation are used for the PDV16 liquids. See **Tables 1** and **2** in the main text. To predict the viscosity for the copolymer liquids, the derived $G(t)$ is used to calculate the simulation-based zero-rate viscosity according to $\eta_{\mathrm{d}} = \int_0^{\infty} G(t)dt$ at $T = 550.0$ K. The results are shown in **Table 1** in the main text. To predict the relaxation modulus for the copolymer gums and compare with the experimental results, the simulation-based $G(t)$ are shifted to the reference temperature $T_0 = -173.0$ K using the experimental shift factor a_{T} according to the WLF relation as in equation S1.

Incoherent scattering function. The incoherent scattering function is used to trace the monomeric motion at a given length scale $\boldsymbol{q}$:

$$S_{\mathrm{inc}}(q,t) = \frac{1}{N}\sum_{j}^{N} \langle \exp\{i\boldsymbol{q} \cdot [\boldsymbol{r}_j(t) - \boldsymbol{r}_j(0)]\} \rangle \qquad \text{eq. S5}$$

Results are plotted in **Figure S20**. The q values of 1.0, 0.14, and 0.08 Å^{-1} correspond to $(2\pi/q)$ the physical dimensions of 6.28, 44.8, and 78.5 Å, respectively. In **Figure S20a**, the segmental relaxation is investigated as the equilibrium bond length is 3.20 Å. As ϕ_{loc} increases, the S_{inc} curves shift rightward, suggesting delayed relaxation. Similar trends are also observed in the $q =$

0.14 and $q = 0.08$ Å^{-1} cases, confirming the delayed relaxation at larger length scale (up to size of a tube segment). However, the characteristic relaxation times are much larger, while the differences between different S_{inc} curves are smaller in these two cases than the $q = 1.0$ Å^{-1} case. The results in **Figures S20b** and **c** support the description of the collective dynamics in the main text.

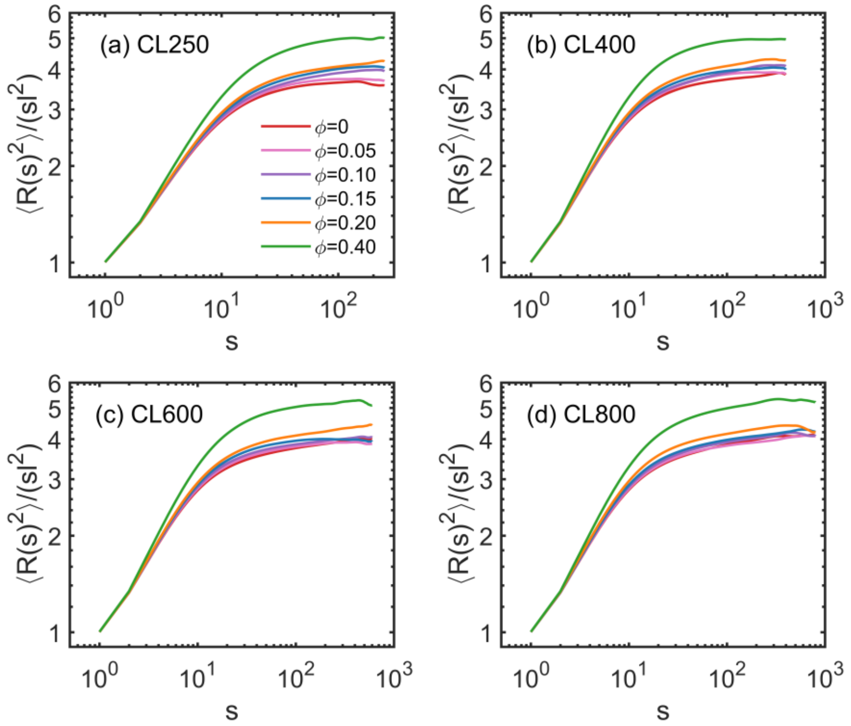

Figure S1. Mean-squared internal distance. (a) to (d) are for the CL250 to C800 systems, respectively. The plateaus of the curves indicate that the configuration is sufficiently equilibrated. The mean-squared end-to-end distance $\langle R_{ee}^2 \rangle$ is also quantified by the last point of each curve.

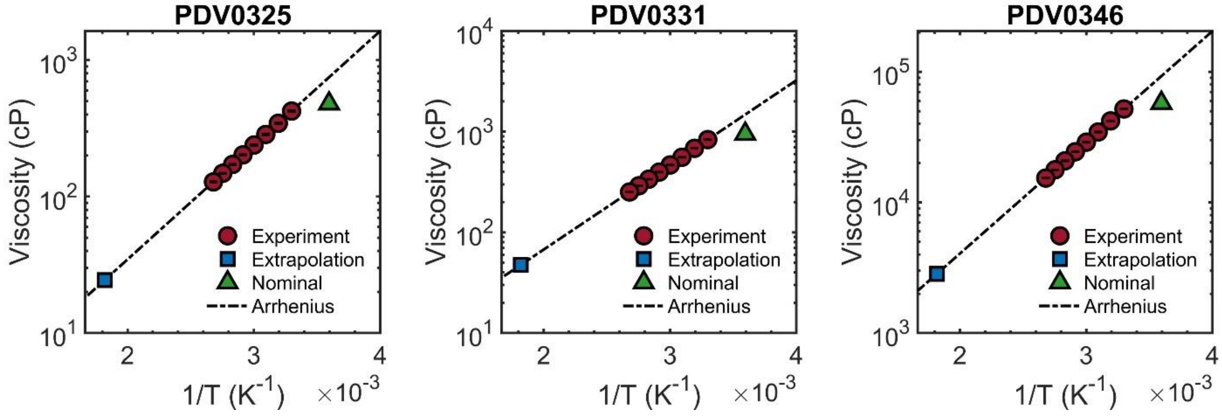

Figure S2. Temperature-dependent dynamic viscosity η_d of the copolymers with $\phi = 0.0325$. The errorbars represent the standard deviation of at least five different measuring values. The measurement was conduct within the linear viscoelascity regiem.

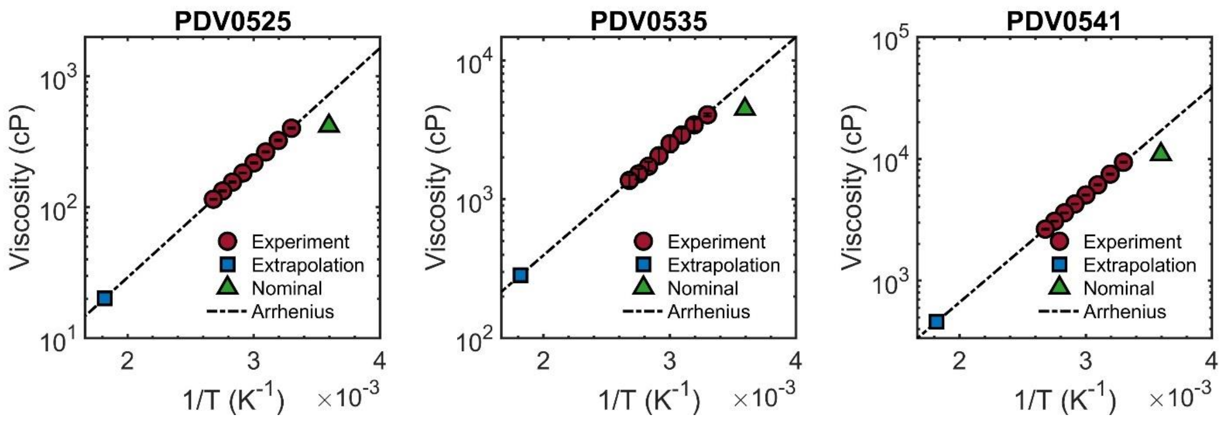

Figure S3. Temperature-dependent dynamic viscosity η_d of the copolymers with $\phi = 0.05$.

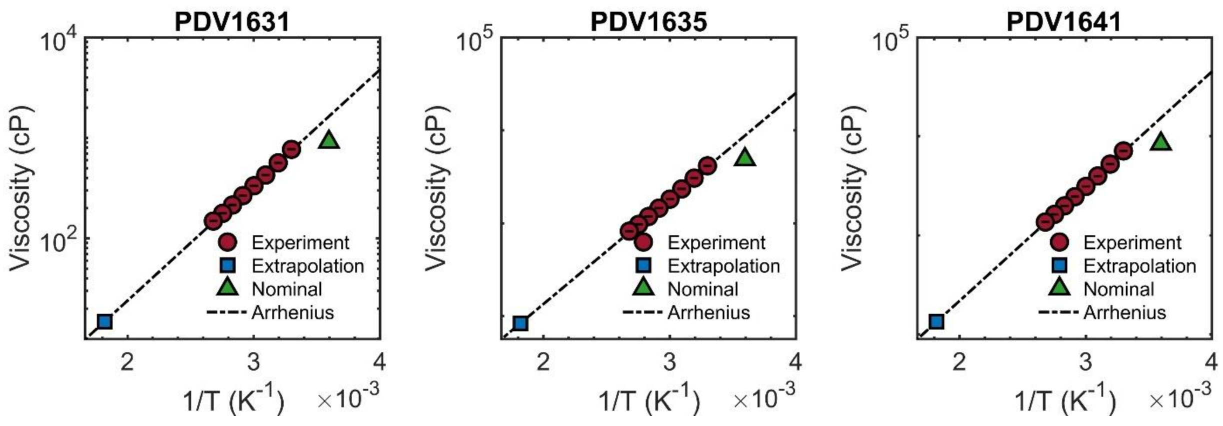

Figure S4. Temperature-dependent dynamic viscosity η_d of the copolymers with $\phi = 0.16$.

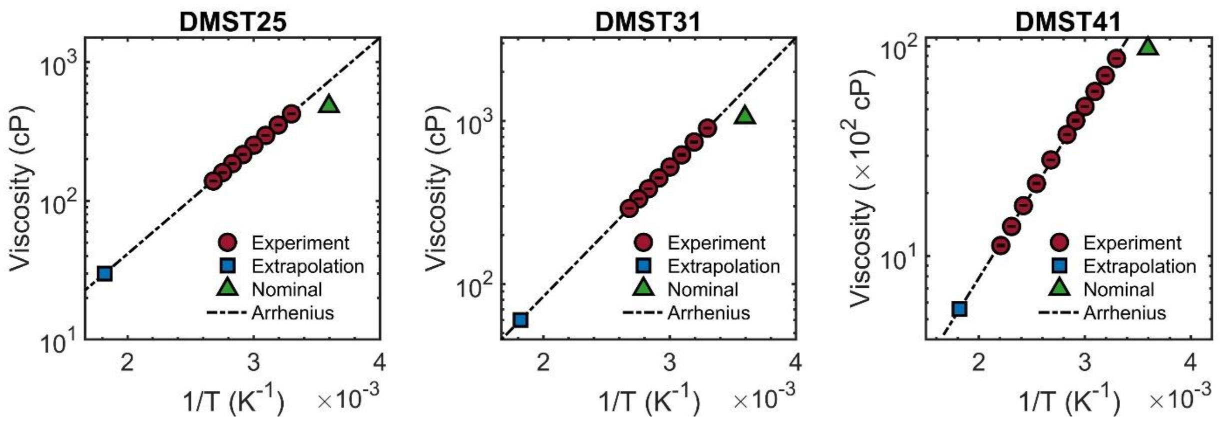

Figure S5. Temperature-dependent dynamic viscosity η_d of the pure PDMS with $\phi = 0.0$.

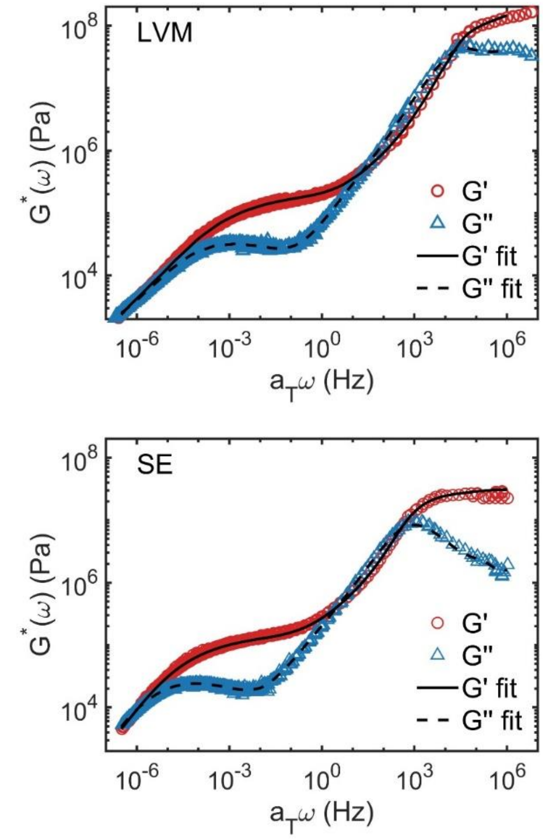

Figure S6. Master curves of the complex modulus $G^*(\omega)$ of the two copolymer gums. The experimental curves were fitted with the generalized Maxwell model with 20 modes. The fitting results are shown in solid and dashed lines.

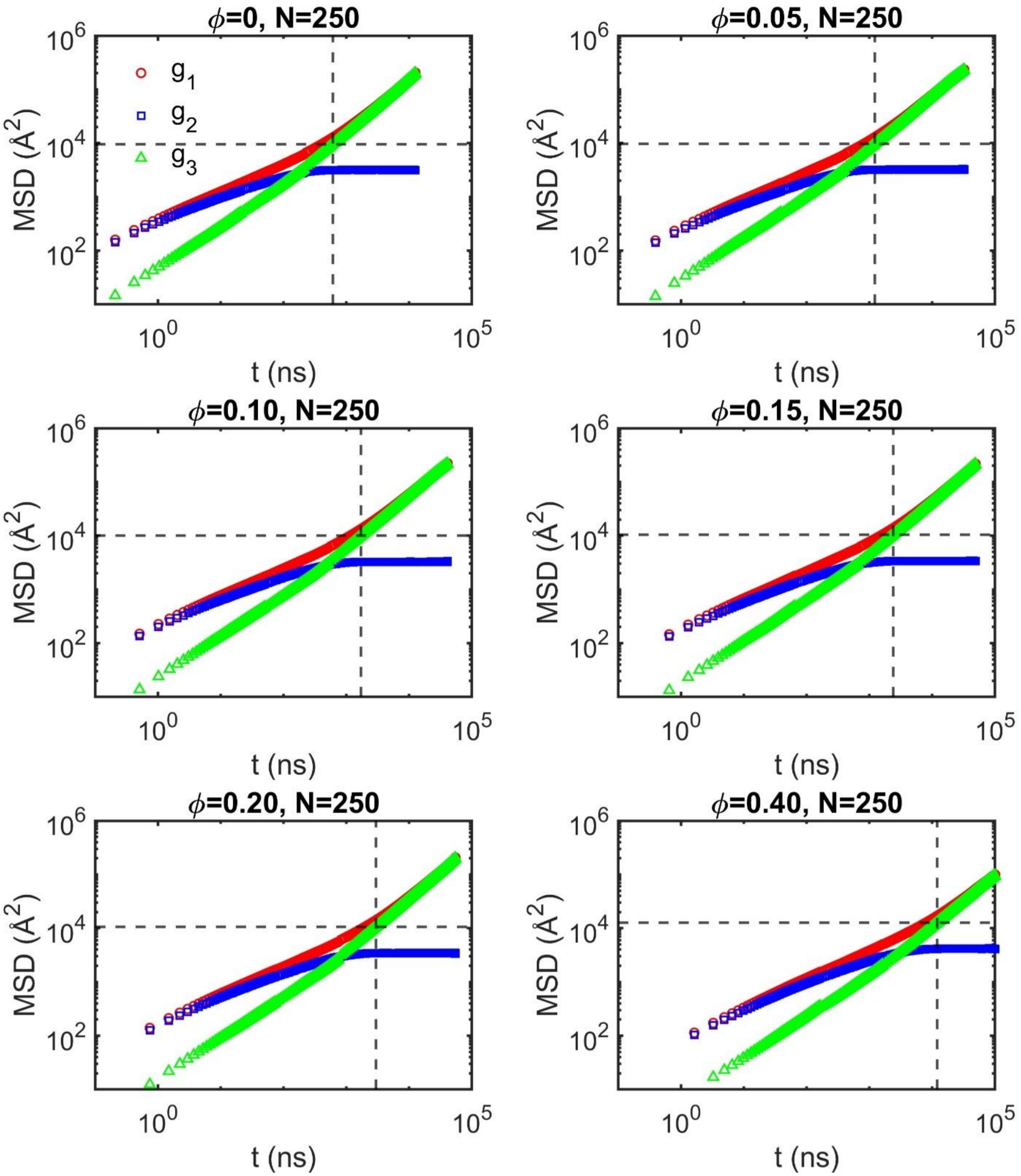

Figure S7. Mean-squared displacement results for the CL250 systems. The marked horizontal dashed line is for the mean-squared end-to-end distance $\langle R_{\mathrm{ee}}^2 \rangle$. It is used to estimate the disentanglement time τ_{d}, marked by the vertical dashed line.

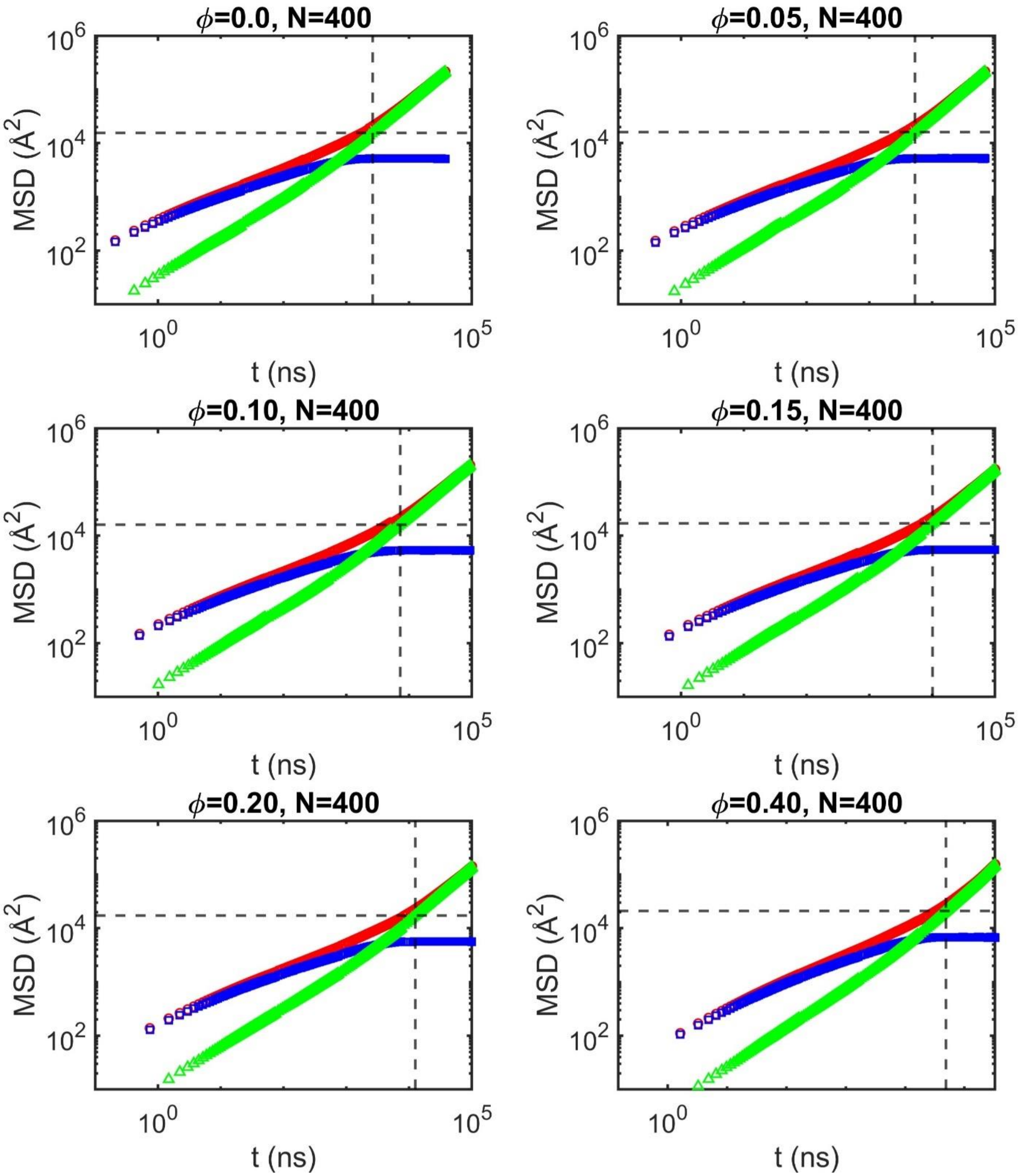

Figure S8. Mean-squared displacement results for the CL400 systems. The full relaxation is indicated by the overlap of the g_1 and g_3 curves and by the plateau of the g_2 curve.

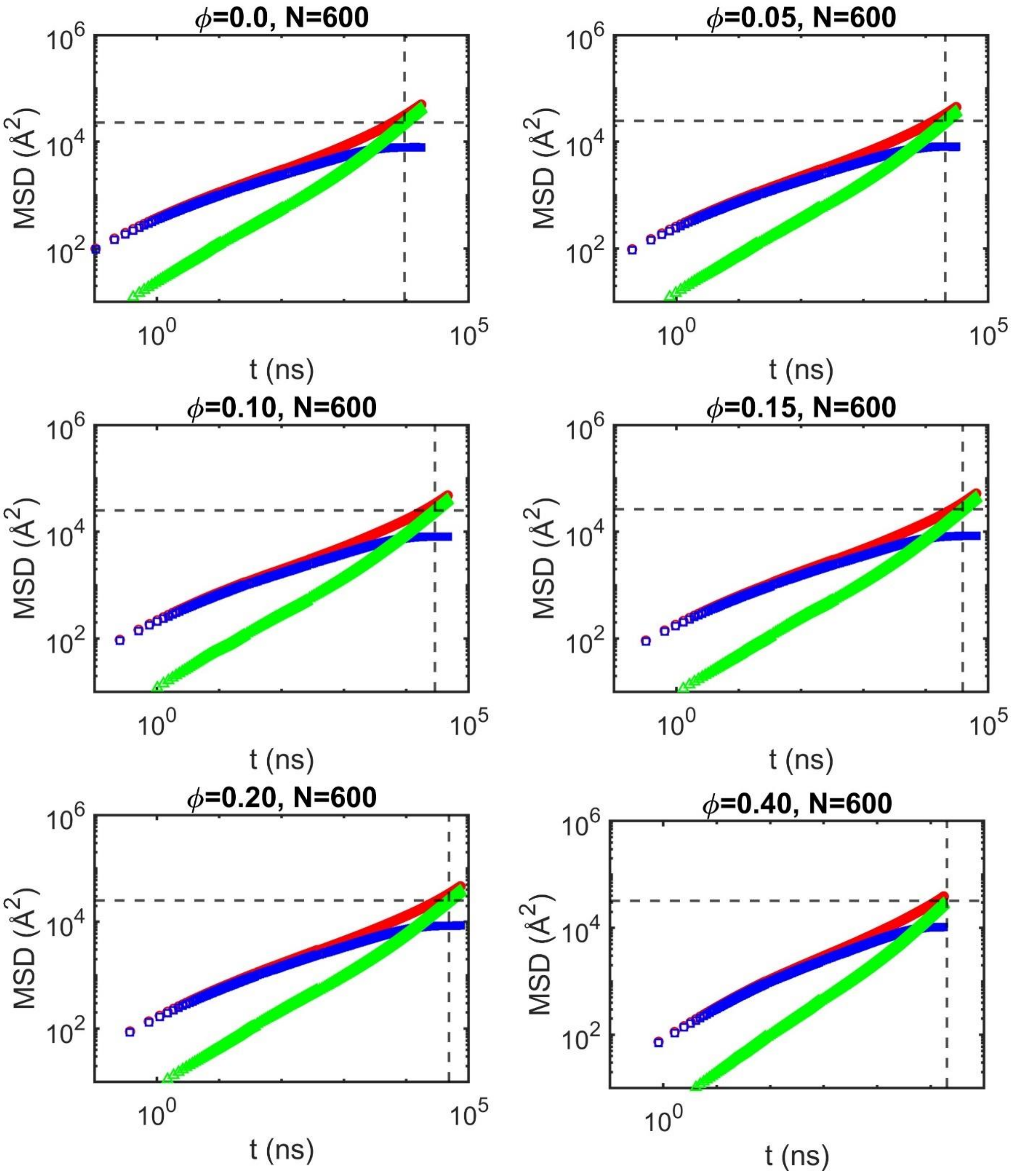

Figure S9. Mean-squared displacement results for the CL600 systems. For the ϕ=0.4 system extrapolation is used to estimate the disentanglement time τ_{d} because the g_3 curve does not intersect with the horizontal dashed line.

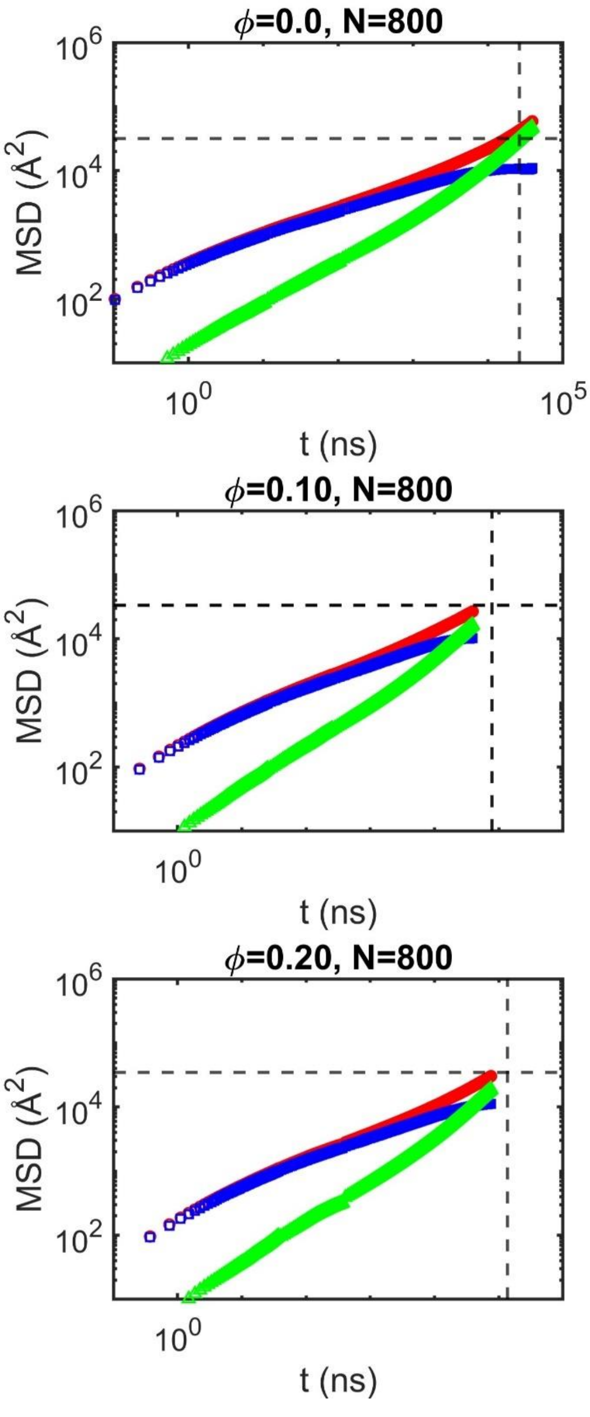

Figure S10. Mean-squared displacement results for the CL800 systems. For the ϕ=0.05 to 0.2 systems extrapolation is used to estimate the disentanglement time τ_{d} because the g_3 curves do not intersect with the horizontal dashed lines.

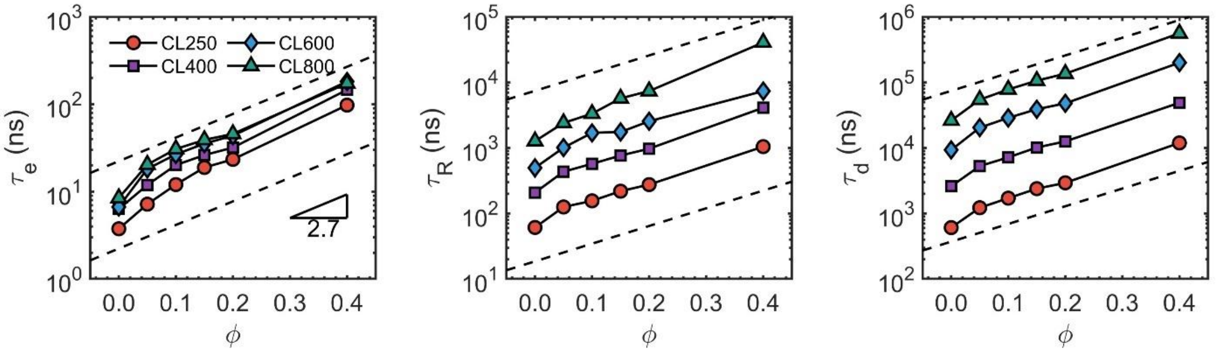

Figure S11. Charaterisic times τ_{e}, τ_{R}, and τ_{d} estimated by the MSD curves. The estimation is based on the change of scaling behaviors of the MSD curves. See main text for details.

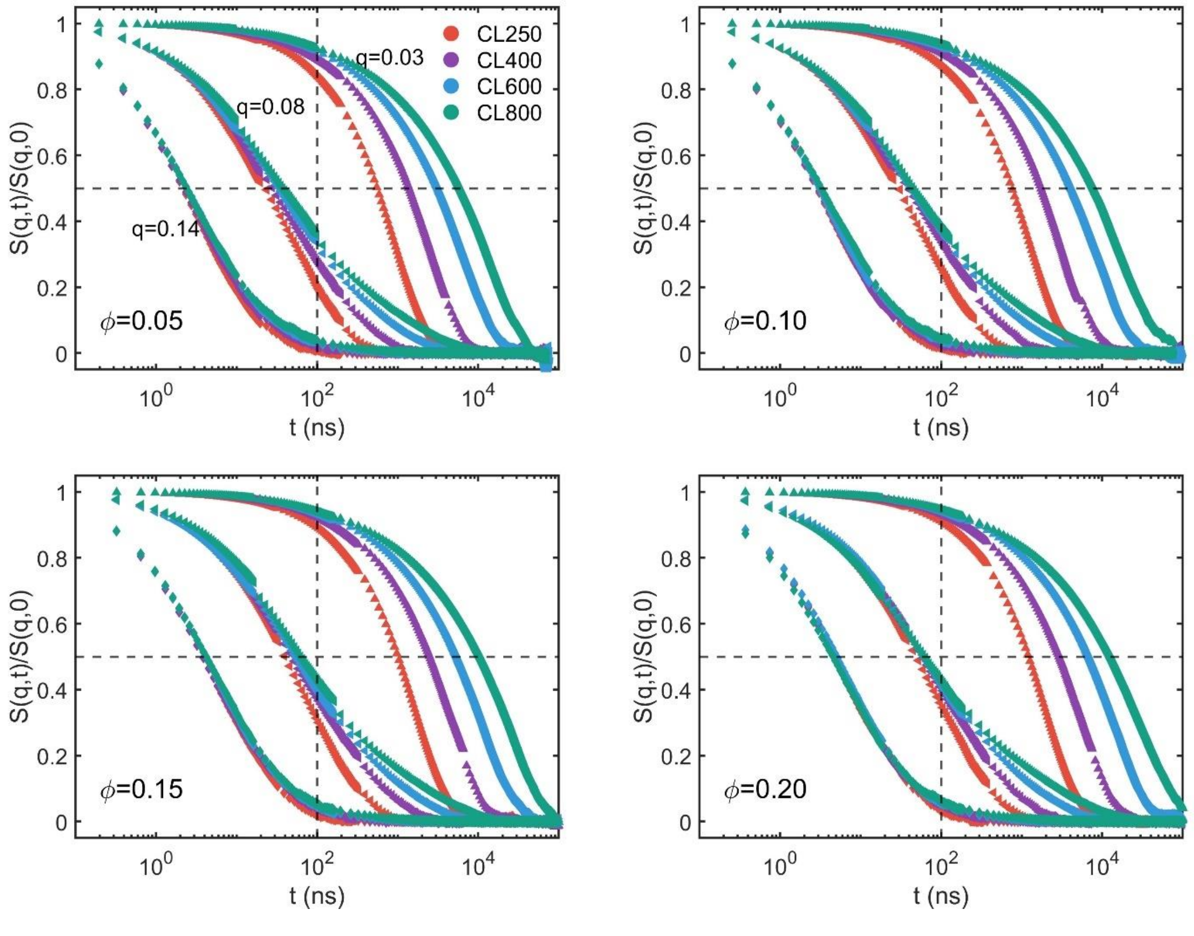

Figure S12. Dynamic structure factor. As $q = 0.14$ Å^{-1} represents a length scale smaller than the tube diameter, there is no constraining effect such that the decay pattern is independent of the chain length N. However, with $q = 0.08$ Å^{-}1 and $q = 0.03$ Å^{-1}, the long-chain systems show prolonged decays, suggesting that the entanglement effect is stronger for the long-chain systems.

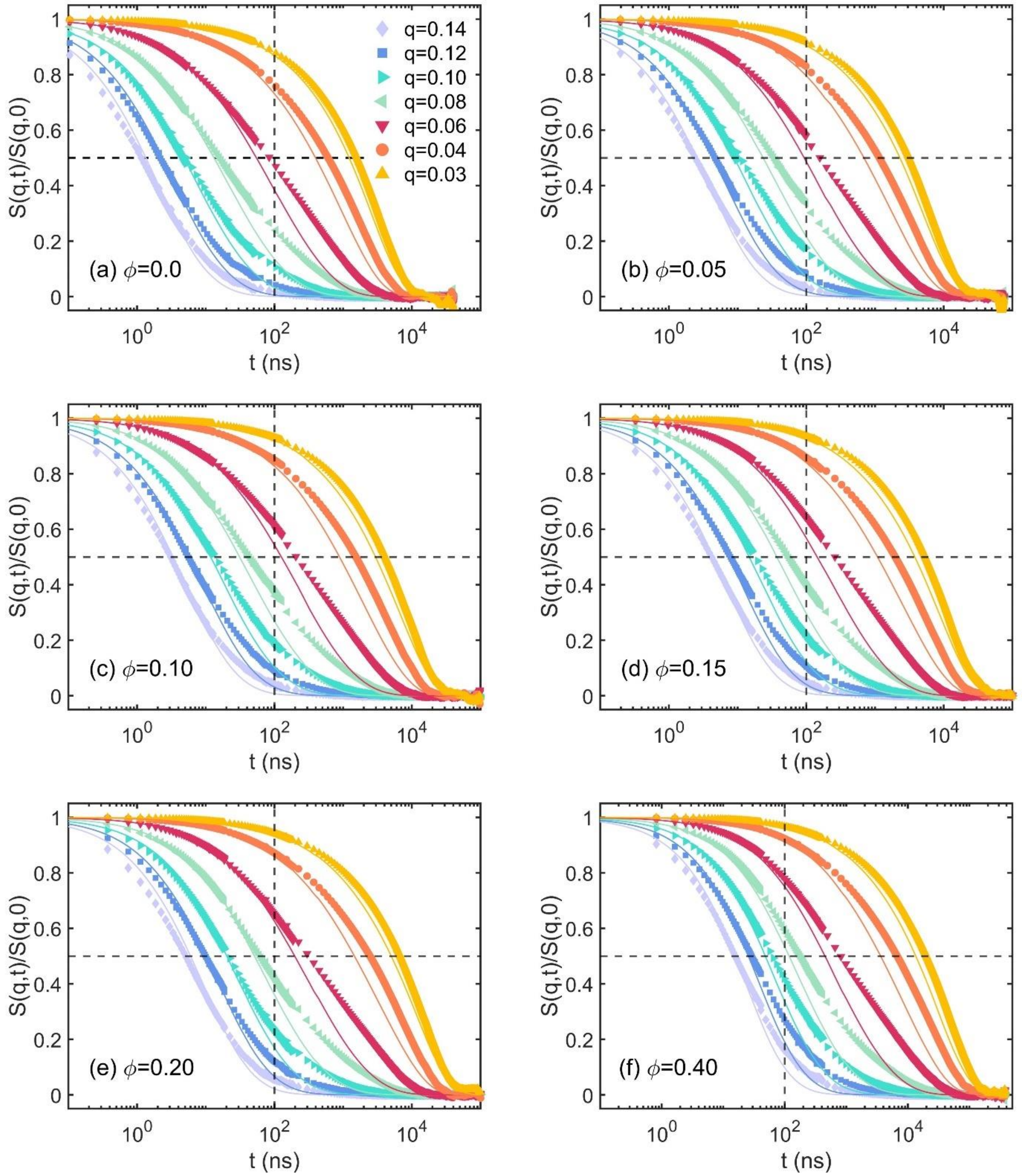

Figure S13. The coherent dynamics structure factor for the CL600 systems. The symbols are the MD simulation results, and the solid lines are the prediction by the Rouse model. While the Rouse model predicts the DSF decay well at the large and the small length scales, it underestimates at the medium length scales at around 100 ns. The amplitude of deviation increases as the molar ratio ϕ increases. These results suggest the entanglement effect at the intermediate and large length scales.

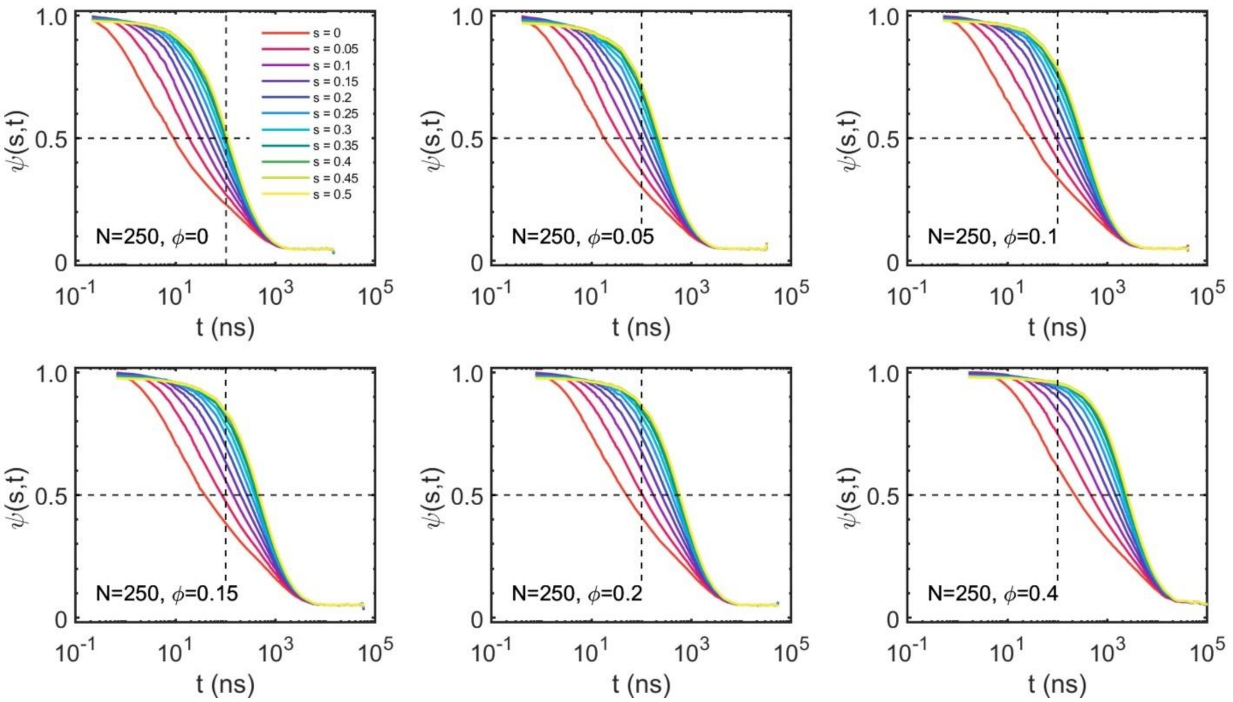

Figure S14. Segment survival probability for the CL250 systems. The dashed lines are for guide to eye. The curves converge to a plateau value of about 0.05 as the full relaxation is reached.

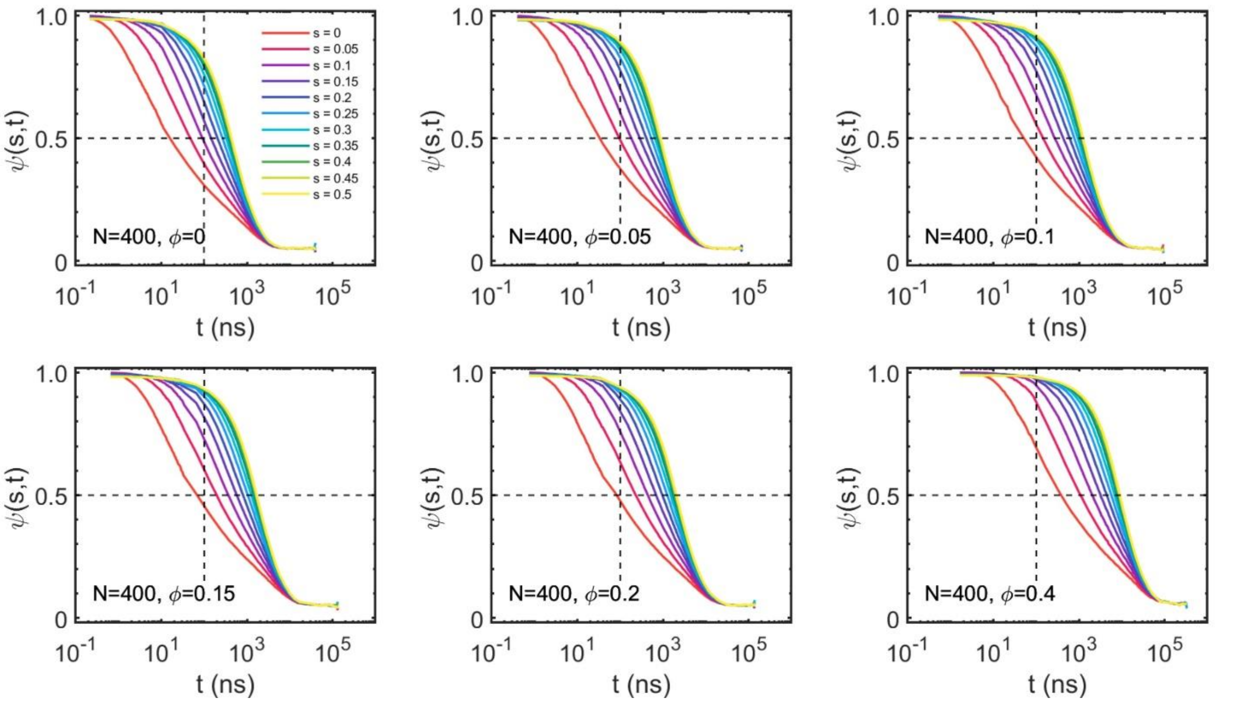

Figure S15. Segment survival probability for the CL400 systems. The dashed lines are for guide to eye. The curves converge to a plateau value of about 0.05 as the full relaxation is reached.

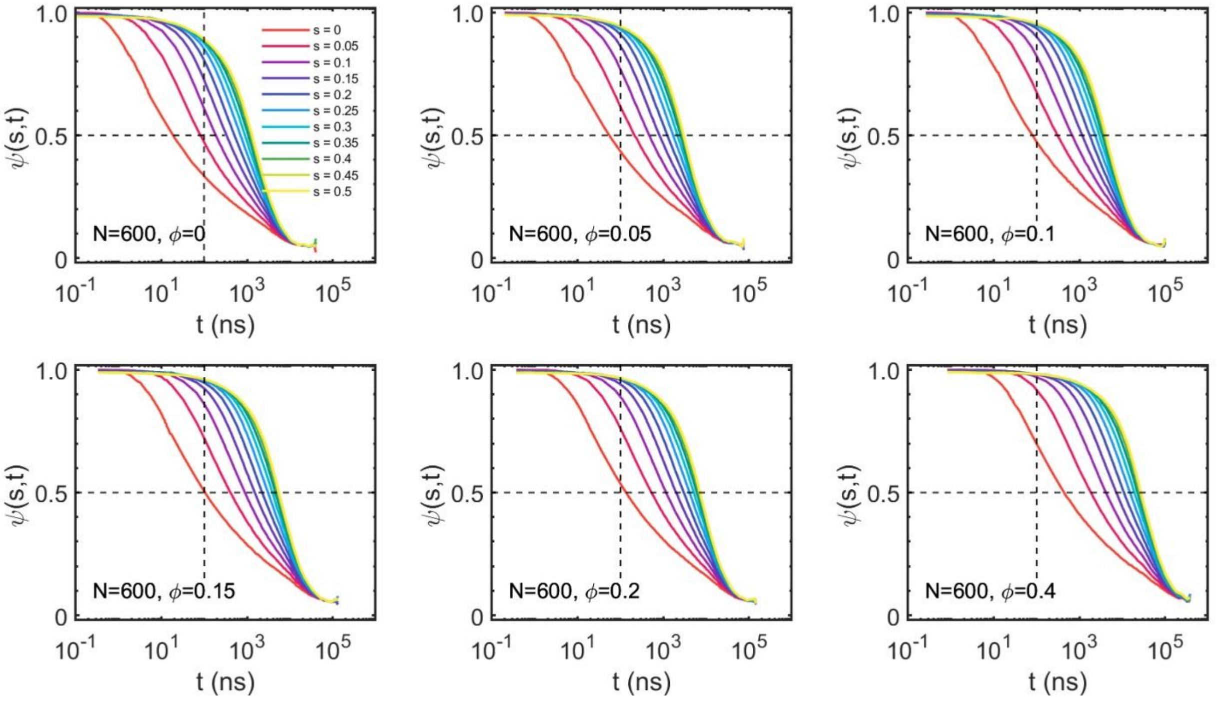

Figure S16. Segment survival probability for the CL600 systems. The dashed lines are for guide to eye. However, the plateau value of 0.05 is not reached for the ϕ=0.4 system.

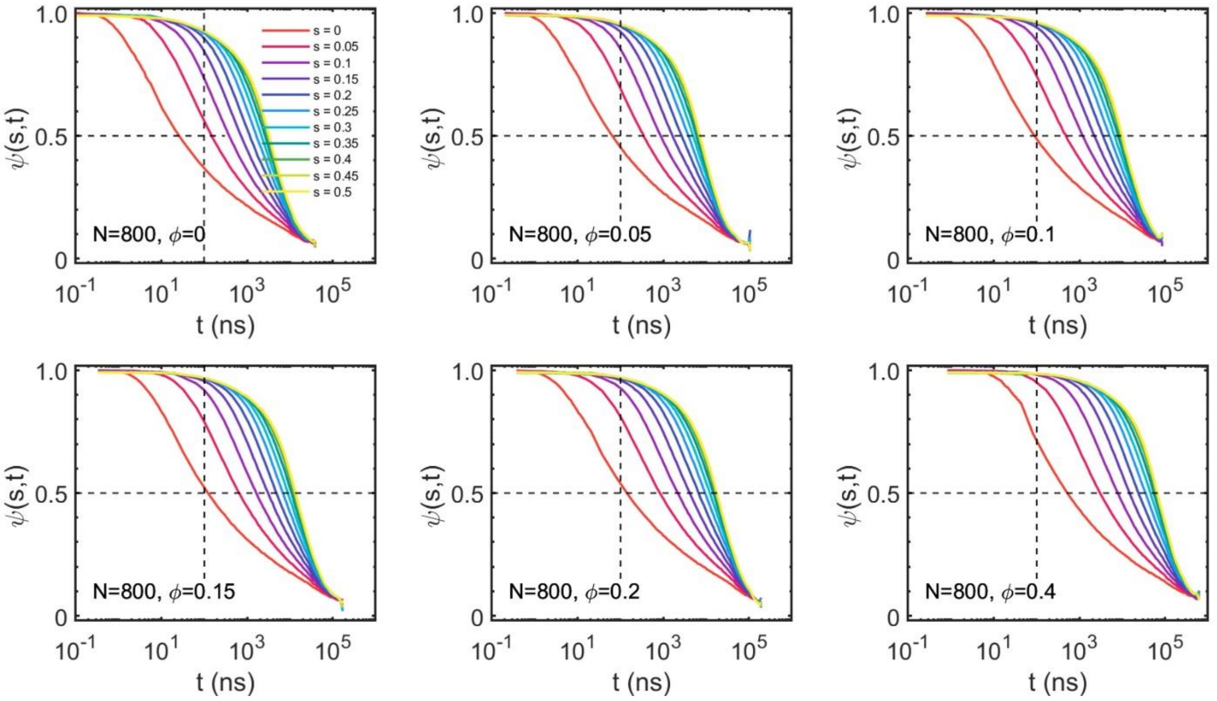

Figure S17. Segment survival probability for the CL800 systems. The terminal plateaus are not as clear as the results of shorter chains, but it is believed that full relaxation is achieved.

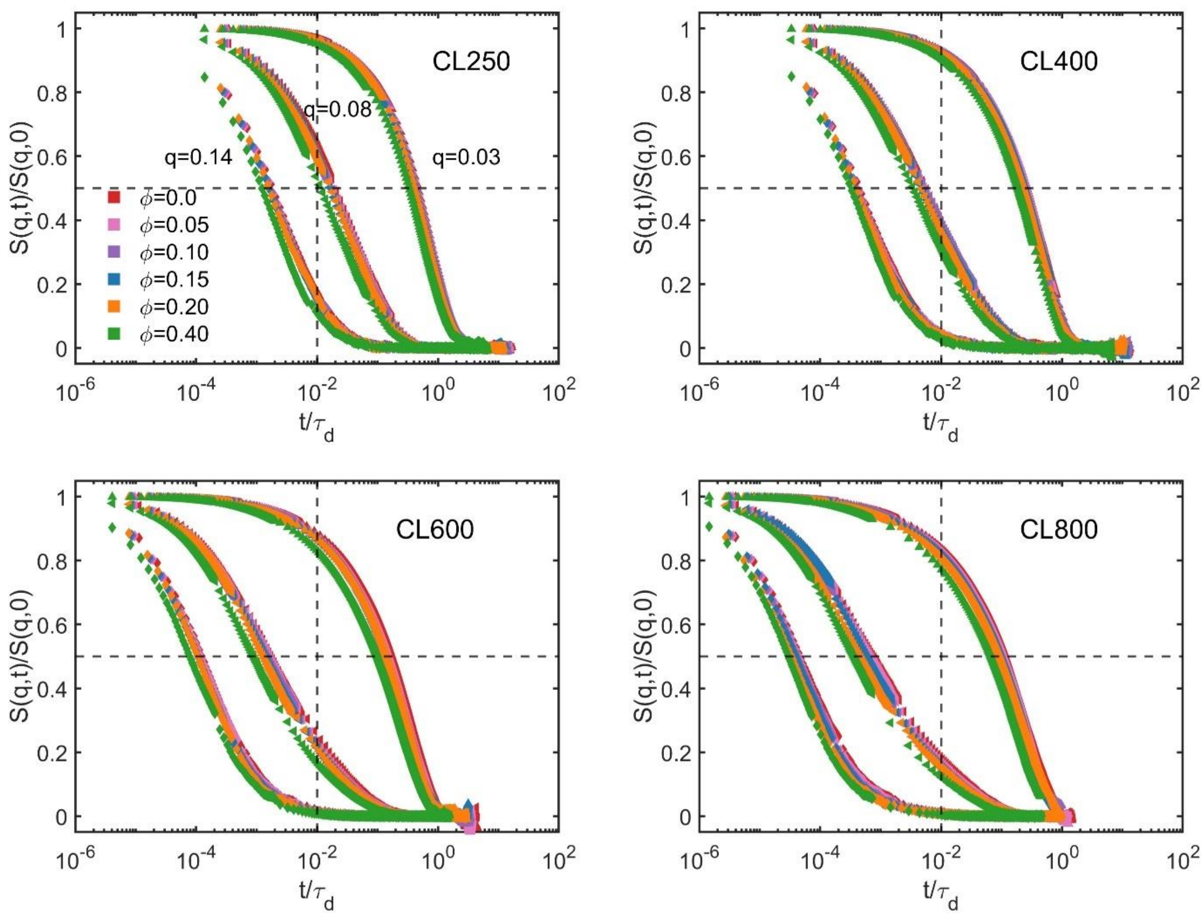

Figure S18. Coherent dynamic structure factor comparison. The DSF decays are rescaled by the respective disentanglement times τ_{d}. The curves collapse to the respective master curves, suggesting the universality of the entangled dynamics. At the chain-level, the molecular relaxation is homogeneous.

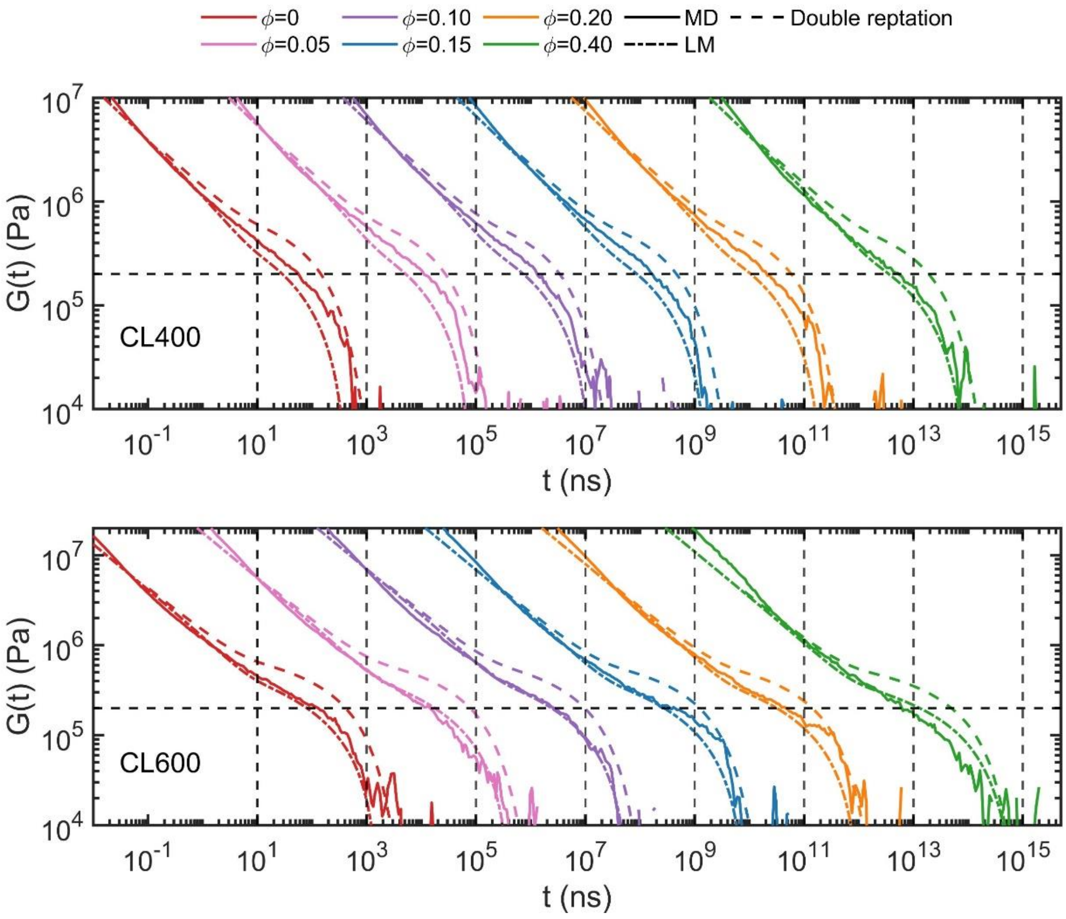

Figure 19. Relaxation modulus for the CL400 and CL600 systems. The solid, dashed, and dot-dashed lines are for the MD result, double-reptation, and Likhtman-McLeish models. The curves are shifted rightward for visual clarity.

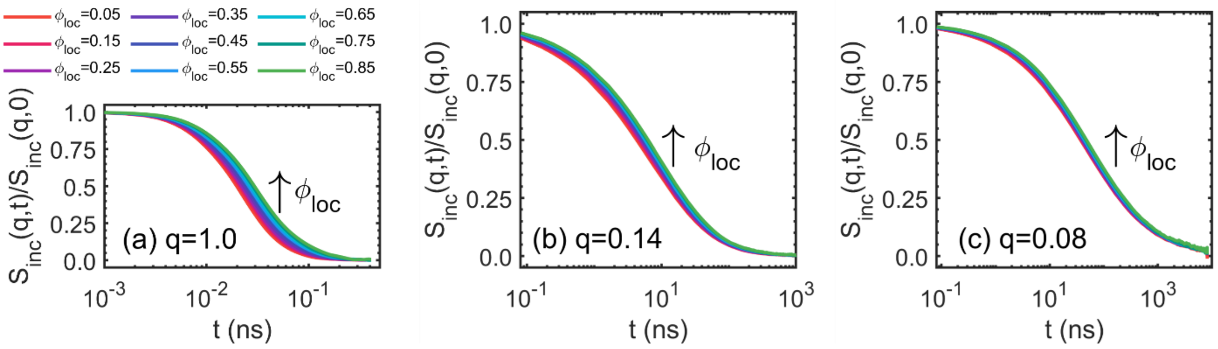

Figure S20. The incoherent dynamics structure factor. All results show that the local relaxation dynamics is slowed down as ϕ_{loc} increases. The higher ϕ_{loc} causes slower relaxation. However, such dynamic heterogeneity is limited and localized to the length scales of segments.

References

(1) Xian, W.; Maiti, A.; Saab, A. P.; Li, Y. Development of a Coarse-Grained Molecular Dynamics Model for Poly (Dimethyl-Co-Diphenyl) Siloxane. *Soft Matter* **2024**, *20* (42), 8480–8492.

(2) Xian, W.; Liu, C.-H.; Kangarlou, B.; Chang, S.-Y.; Wu, C.; Cao, Y.; Sun, L.; Ma, A. W. K.; Nieh, M.-P.; Maiti, A.; others. Effect of Diphenyl Content on Viscoelasticity of Poly (Dimethyl-Co-Diphenyl) Siloxane Melt and Network. *ACS Appl Polym Mater* **2023**, *5* (3), 1915–1925.

(3) Padding, J. T.; Briels, W. J. Zero-Shear Stress Relaxation and Long Time Dynamics of a Linear Polyethylene Melt: A Test of Rouse Theory. *J Chem Phys* **2001**, *114* (19), 8685–8693.

(4) Xian, W.; He, J.; Maiti, A.; Saab, A. P.; Li, Y. Investigating Structure and Dynamics of Unentangled Poly (Dimethyl-Co-Diphenyl) Siloxane via Molecular Dynamics Simulation. *Soft Matter* **2023**, *19* (23), 4265–4276.

(5) Padding, J. T.; Briels, W. J. Time and Length Scales of Polymer Melts Studied by Coarse-Grained Molecular Dynamics Simulations. *J Chem Phys* **2002**, *117* (2), 925–943.

(6) Kröger, M.; Dietz, J. D.; Hoy, R. S.; Luap, C. The Z1+ Package: Shortest Multiple Disconnected Path for the Analysis of Entanglements in Macromolecular Systems. *Comput Phys Commun* **2023**, *283*, 108567.

(7) Stephanou, P. S.; Baig, C.; Tsolou, G.; Mavrantzas, V. G.; Kröger, M. Quantifying Chain Reptation in Entangled Polymer Melts: Topological and Dynamical Mapping of Atomistic Simulation Results onto the Tube Model. *J Chem Phys* **2010**, *132* (12).

(8) Des Cloizeaux, J. Double Reptation vs. Simple Reptation in Polymer Melts. *Europhys Lett* **1988**, *5* (5), 437.

(9) Des Cloizeaux, J. Relaxation of Entangled Polymers in Melts. *Macromolecules* **1990**, *23* (17), 3992–4006.

(10) Matsumiya, Y.; Watanabe, H.; Osaki, K. Comparison of Dielectric and Viscoelastic Relaxation Functions of Cis-Polyisoprenes: Test of Tube Dilation Molecular Picture. *Macromolecules* **2000**, *33* (2), 499–506.

(11) Watanabe, H. Dielectric Relaxation of Type-A Polymers in Melts and Solutions. *Macromol Rapid Commun* **2001**, *22* (3), 127–175.

(12) Behbahani, A. F.; Schmid, F. Relaxation Dynamics of Entangled Linear Polymer Melts via Molecular Dynamics Simulations. *Macromolecules* **2024**, *58* (1), 767–786.

(13) Likhtman, A. E.; McLeish, T. C. B. Quantitative Theory for Linear Dynamics of Linear Entangled Polymers. *Macromolecules* **2002**, *35* (16), 6332–6343.

(14) Ram\'\irez, J.; Sukumaran, S. K.; Vorselaars, B.; Likhtman, A. E. Efficient on the Fly Calculation of Time Correlation Functions in Computer Simulations. *J Chem Phys* **2010**, *133* (15).
